# Higher order synthetic lethals are keys to minimize cancer treatment effects on non-tumor cells

**DOI:** 10.1371/journal.pone.0342199

**Published:** 2026-04-15

**Authors:** Mehdi Dehghan Manshadi, Payam Setoodeh, Amin Ramezani, Amin Reza Rajabzadeh, Habil Zare

**Affiliations:** 1 Department of Oncology-Pathology, Karolinska Institutet, Stockholm, Sweden; 2 Algorithmic Dynamics Lab, Center for Molecular Medicine, Karolinska University Hospital, Stockholm, Sweden; 3 Department of Chemical Engineering, School of Chemical, Petroleum and Gas Engineering, Shiraz University, Shiraz, Iran; 4 W Booth School of Engineering Practice and Technology, McMaster University, Hamilton, Ontario, Canada; 5 Shiraz Institute for Cancer Research, School of Medicine, Shiraz University of Medical Sciences, Shiraz, Iran; 6 Department of Medical Biotechnology, School of Advanced Medical Sciences and Technologies, Shiraz University of Medical Sciences, Shiraz, Iran; 7 School of Biomedical Engineering, McMaster University, Hamilton, Ontario, Canada; 8 Haba Businesses, San Antonio, Texas, United States of America; Federal University Dutse, NIGERIA

## Abstract

Metabolic rewiring in cancer cells facilitates the supply of essential precursors for uncontrolled tumor growth. Exploring these cancer-specific metabolic alterations offers potential selective therapeutic strategies. However, targeting a single essential gene in cancer treatment often faces challenges, including resistance, lack of targetable oncogenes, and potential harm to non-tumor cells. Targeting multiple genes has been proposed as a solution to overcome these issues, e.g., a synthetic lethal (SL) set, defined as a minimal combination of non-lethal genetic perturbations that lead to cell death. This study theoretically examined the potential of SL sets to identify selective drug targets across 13 cancer types and the corresponding non-tumor tissues, utilizing context-specific genome-scale metabolic models. To ensure the minimization of therapeutic side effects, this work introduced the concept of *strictly-selective* drug targets (SSDTs) and the lack of harmful effects of the identified targets in all 13 different non-tumor tissues was meticulously verified. Accordingly, for 13 types of cancers, over 500 SSDTs were identified, predominantly including higher-order SL sets with more than two targets in each set. Interestingly, for specific cancers where single essential or SL genes could not provide viable therapeutic solutions, SSDTs were provided by higher-order SL sets. Thus, for the first time, this study demonstrates that leveraging higher-order SL sets may offer promising strictly selective therapeutic solutions. Furthermore, nine quadruple SSDTs were identified, which commonly target five different cancers without harming any of the 13 non-tumor tissues. Further experimental validation of these findings is essential to identify the most promising treatment candidates for future clinical studies/applications.

## Introduction

Metabolic rewiring leads to distinct alterations in flux distributions across various pathways in cancer cells. Otto Warburg was the first to highlight these metabolic variations, demonstrating that cancer cells preferentially rely on aerobic glycolysis rather than oxidative phosphorylation [1]. This phenomenon, known as the Warburg effect, illustrates how these metabolic alterations in cancer cells support the increased demand for fatty acids, nucleotides, and amino acids [2]. Extensive research has focused on aerobic glycolysis to better understand cancer cell metabolism [3–13].

In addition to the Warburg effect, numerous other metabolic alterations have been reported across different cancer types. One such alteration is the upregulation of the glutaminolysis pathway, which, together with enhanced glycolysis, supplies both energy and biosynthetic precursors to sustain elevated proliferation rates [14–16]. Furthermore, the literature indicates the deregulation of other pathways, such as the lipid synthesis pathway [17], the branched-chain amino acid metabolism, the serine synthesis pathway [18], and the pentose phosphate pathway [19]. Metabolic rewiring in cancer cells also presents unique opportunities for immune evasion [20], apoptosis disruption [21], and the utilization of metabolic byproducts such as ammonia, ketone bodies, acetate, and lactate [18].

Characterization and identification of the mentioned cancer-specific metabolic alterations pave the way for developing selective therapeutic strategies that exclusively affect cancer cells without harming non-tumor tissues [22–24]. Genome-scale metabolic models (GEMs), which represent comprehensive mathematical reconstructions of cellular metabolism, have emerged as powerful tools for studying metabolic rewiring at the systems level [25–27]. Consequently, various computational approaches have been developed to use these models for identifying metabolic cancer drug targets [28,29]. Folger et al. [30] provided one of the earliest studies in the field of drug target identification using GEMs. The authors provided a unified model using expression data from different cancers and found 52 drug targets, 21 of which corresponded to experimentally validated or known anticancer drugs. To generate context- or patient-specific GEMs, a number of approaches have been developed and applied to identify different cancer therapeutics [31]. Yizhak et al. [32] constructed more than 280 healthy and cancerous models using PRIME (personalized reconstruction of metabolic models). They applied this set of cell-specific models to predict selective drug targets, experimentally validating the top predicted target, *MLYCD*, and investigating the depletion effects of this gene. In another study, Gatto et al. [33] used the INIT algorithm [34] to develop GEMs for clear cell renal cell carcinoma (ccRCC) and successfully identified five metabolic genes as selective drug targets, predicted to be dispensable in non-tumor cell metabolism. Barrena et al. [35] extended the gMCS approach by integrating linear regulatory pathways with metabolic models using Human1 and regulatory databases. They identified new essential genes and synthetic lethal (SL) pairs in cancer cell lines. Larsson et al. [36] used a generic GEM to identify five selective essential genes for glioblastoma, with *in vitro* or *in vivo* evidence of the essentiality of 4 out of the 5 being reported in the literature. Pacheco et al. [37] introduced the rFASTCORMICS algorithm and constructed 10,005 GEMs for 13 different types of cancers. The authors performed a single gene essentiality analysis to identify selective drug targets and used colorectal cancer as a successful experimental test case for drug repurposing of mimosine, ketoconazole, and naftifin.

Despite valuable single-drug targets being identified using the mentioned approaches, based on the literature, targeting only one gene presents several challenges [38]. The survival dependency of a tumor on an oncogene or oncogenic pathway is a phenomenon known as oncogene addiction. Currently, this phenomenon forms the basis of most genotype-targeted cancer therapeutics. However, gain-of-function oncogenes are not targetable in all tumors, resulting in common resistance to the administered therapy [39]. Furthermore, identifying unmarked oncogenes, the cancer-specific driver genes with no evidence of genetic alterations, is challenging. To address these limitations, the concept of synthetic lethality has been proposed [39–41]. Synthetic lethality describes a pair of genes for which individual perturbations are non-lethal, whereas their simultaneous disruption results in cell death. This principle offers the potential to selectively target cancer cells by exploiting tumor-specific genetic or metabolic vulnerabilities [42]. Moreover, SL sets provide opportunities to target cancers with pathway alterations that are otherwise considered undruggable [43]. Although the capabilities of SLs for providing selective drug targets are mentioned in the literature, relatively few studies have systematically explored their application. In this context, Frezza et al. [44] investigated fumarate hydratase Fh1-deficient kidney cells. The researchers constructed context-specific genome-scale metabolic models (GEMs) for cell lines with and without Fh1, using gene expression data. They predicted gene knockouts that were lethal to Fh1-deficient cells but did not affect non-tumor cells. The analysis highlighted reactions—primarily in the heme metabolism pathway—as synthetically lethal with Fh1 deletion. In another study, Zhang et al. [45] performed an exhaustive search to identify 44 double and 95 triple selective drug targets for hepatocellular carcinoma (HCC) using a generic GEM from Argen et al. [46].

In addition to mechanistic GEM-based strategies, several computational and machine-learning approaches have been developed to predict SL interactions from large-scale genomic and transcriptomic data. These include network-based models, matrix-completion frameworks, and emerging deep-learning architectures [47]. While such methods offer complementary, data-driven perspectives, their predictive performance remains variable, especially for multiple-gene intervention, and further methodological refinement is needed before they can reliably guide SL discovery on their own [48].

Although synthetic lethality is considered a more robust strategy for identifying selective drug targets compared to single-gene targeting, to the best of our knowledge, no systematic study has yet demonstrated this advantage. Therefore, this work aims to identify SL gene sets predicted by analyses of context-specific genome-scale metabolic models to determine the capabilities of the concept of synthetic lethality for the identification of *strictly-selective* drug targets. Here, the term “*strictly-selective*” refers to targets that affect cancer cells while minimizing damage not only to the corresponding non-tumor tissue but also to non-tumor tissues across different organs. To achieve this objective, context-specific models were constructed for 13 different pairs of cancerous and non-tumor tissues using the rFASTCORMICS algorithm. The names and abbreviations of the studied cancers are listed in Table 1.

**Table 1:** TCGA abbreviations of the cancers studied in this paper.

TCGA name	Expanded name
COAD	Colon adenocarcinoma
BRCA	Bladder Urothelial carcinoma
LUAD	Lung adenocarcinoma
LIHC	Liver hepatocellular carcinoma
LUSC	Lung squamous cell carcinoma
UCEC	Uterine Corpus endometrial carcinoma
HNSC	Head and neck squamous cell carcinoma
STAD	Stomach adenocarcinoma
KIRC	Kidney renal clear cell carcinoma
PRAD	Prostate adenocarcinoma
KICH	Kidney chromophobe
THCA	Thyroid carcinoma
KIRP	Kidney renal papillary cell carcinoma

For these models, essential genes, SL pairs, and higher-order SL sets (comprising up to four genes), were identified. Subsequently, potential *strictly-selective* drug targets were determined. The results showed that when a few or even no essential genes can be targeted due to causing side effects for different non-tumor cells in the human body, higher-order SL sets represent a key strategy for identifying *strictly-selective* drug targets that discriminate solely against cancerous tissue(s). Accordingly, in addition to the identification of several *strictly-selective* potential drug targets for 13 cancers, for the first time, this study successfully showed the capabilities of the concept of synthetic lethality for providing strictly selective drug targets for cancers applying genome-scale metabolic modeling and *in-silico* experimentations/analyses.

## Methods

### Reconstruction of context-specific models

In this study, 26 context-specific models, consisting of 13 generic control models and 13 generic cancer models, were reconstructed using rFASTCORMICS [37], a promising tool for generating accurate models capable of predicting the outcomes of gene knockout strategies. The Recon 2.04 [49] genome-scale reconstruction (RRID:SCR_006345) was chosen as the reference model, and the RNA-seq data (GSE62944) [50] were retrieved from the TCGA Research Network (http://cancergenome.nih.gov/). The assignment of samples to cancerous and non-tumor tissues was based on their pathology information available from TCGA. This process could not be randomized. Also, power analysis was not applicable here because we used all existing data.

While arbitrary thresholds for RNA-seq data can substantially influence model precision in classifying expressed and non-expressed genes [51], rFASTCORMICS adopts an approach proposed by Hart et al. [52] to determine active genes. Specifically, rFASTCORMICS generates a density plot for each sample based on its log2-transformed FPKM (Fragments per Kilobase of transcript per Million mapped reads) values. Subsequently, a Gaussian curve is fitted to the right half of the main peak (expression curve). The mean value of this curve is utilized as the expression threshold. Then, the obtained curve is subtracted from the density curve, and another Gaussian curve is fitted to this part of the density curve (inexpression curve).

rFASTCORMICS uses the mean value (μ) and standard deviation (σ) of the right-hand Gaussian curve to convert log2-transformed FPKM values to the zFPKM values.


$$\text{zFPKM }=\frac{log\text{2}(FPKM)\;-\;\mu }{\sigma }$$ (1)

The mean of the inexpression curve was chosen as a threshold if it was above −3 zFPKM; otherwise, −3 was taken, and genes with lower zFPKM values were considered inactive with a score of −1. Conversely, 0 zFPKM was chosen as the expression threshold, and genes with higher zFPKM values were considered active with a score of +1. Genes with zFPKM values between these two thresholds were considered to have an *unknown expression status* with a score of 0. Finally, using gene-protein-reaction (GPR) rules, expression scores for genes were mapped to corresponding reactions, and bounds for inactive reactions were set to zero.

### Extracting SL sets

Extracting SL gene pairs from reconstructed models is not computationally challenging. However, extending the synthetic lethality principle to gene sets with more than two genes and attempting to identify higher-order SL sets (HOSLs) presents significant computational challenges. As the number of genes in an SL set increases, the corresponding search space grows exponentially, making the search process extremely time-consuming. Therefore, an algorithm is required to explore this expanding search space efficiently. In this study, Rapid-SL [53] was applied to identify HOSLs up to quadruples (four genes in a set) for 13 cancerous models. Alternative methods for determining all HOSLs, include gMCS [54] and Fast-SL [55], although these may not be as computationally efficient as Rapid-SL in practice.

### *Strict selectivity* criteria

**Fig 1:**
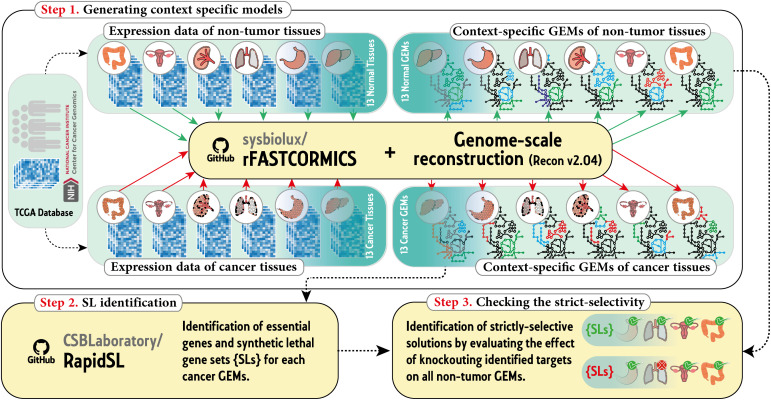
Overview of the workflow followed in this study.

The primary goal of this work was to identify *strictly-selective* potential drug targets. In this study, *strictly-selective* drug targets refer to essential genes or SL gene sets. These targets aim to reduce the growth rate of cancer cells without adversely affecting non-tumor tissues across other organs. To mathematically describe these criteria, certain thresholds were used. Here, a gene (or gene set) was considered lethal (or synthetic lethal) if its knockout reduced the growth rate of cancer cells by over 50% [37]. Furthermore, not affecting non-tumor tissues was defined as causing less than a 10% [37] reduction in the growth rate of non-tumor tissues when the gene (or gene set) was knocked out from the corresponding models. Fig 1 shows a schematic overview of the procedure used in this work.

## Results

This study identified essential genes and SL gene sets for 13 context-specific genome-scale models of different cancers. Additionally, besides identifying SL genes, this study successfully identified triple and quadruple SL gene sets, referred to as higher-order SL sets. The list of *strictly-selective* drug targets obtained in this work is provided in the S1 File. We did not apply druggability filtering when identifying SSDTs. Some strictly selective vulnerabilities involve genes that are not currently tractable by small-molecule inhibitors (e.g., RPE). We chose to report the full spectrum of model-derived vulnerabilities because non-druggable genes may still be useful for biomarker development, pathway dissection, synthetic-lethality sensitization, or future therapeutic strategies that extend beyond conventional small-molecule targeting. Therefore, the presence of non-druggable genes reflects our intention to provide an unbiased and comprehensive map of strictly selective metabolic vulnerabilities.

### Cases with essential genes as *strictly-selective* solutions

Here, the reported results are explained using colon cancer as an example. The number of essential genes and SL sets obtained for the COAD GEM is reported in Table 2. This table illustrates how the number of solutions decreased when their selectivity was tested using the GEM of the non-tumor tissue of the colon.

**Table 2:** Number of essential and SL solutions identified for colon cancer.

	Single essentials	Double SLs	Triple SLs	Quadruple SLs
**Lethal for cancer cells**	76	64	145	416
**Selective for non-tumor colon cells**	12	26	57	135
**Percentage of selective solutions (%)**	15.8	40.1	39.3	32.5

Although the solutions obtained in this step are potentially selective for cancerous colon tissue cells, there remains a possibility of affecting other non-tumor tissues. Therefore, the effect of the remaining solutions on other non-tumor tissues was examined step by step by considering the selectivity criteria for other non-tumor cells, such as breast, lung, etc. By considering each additional non-tumor tissue, some potential solutions may be found to be non-selective and omitted from the list of potential selective drug targets. When considering all 13 non-tumor tissues, many candidate solutions were excluded. As a result, *SLC38A3*, a member of the transport of inorganic cations/anions and amino acids/oligopeptides pathway, has been identified as a single *strictly-*selective drug target. In comparison to *SQLE* proposed by Pacheco et al. [37], which has side effects on other non-tumor tissues, such as the liver or kidney, *SLC38A3* is expected to have minimal side effects on all 13 non-tumor tissues. Asparagine (DB00174) is one of the compounds reported to inhibit *SLC38A3* and may serve as a candidate for validating this *strictly-selective* drug target.

Although no double *strictly-selective* SL set was found, two three-membered SL sets were identified for COAD. The first set includes *RPE*, *HSD11B2*, and *G6PD*. Two of these genes also appear in the second SL set, which includes *RPE*, *HSD11B2*, and *PGLS*. These genes are functionally related. Specifically, *RPE*, *G6PD*, and *PGLS* contribute to the pentose phosphate pathway and the gene *HSD11B2* is related to the glucocorticoid biosynthesis pathway. Various drugs are reported to have effects on *G6PD*, *PGLS*, and *HSD11B2* (see S2 File); however, *RPE* is not listed as a target for any drugs on DrugBank [56]. Because all genes in the set of *RPE*, *G6PD*, and *HSD11B2* are reported to have fold changes greater than 1 [57], we propose this lethal set as a suitable candidate for further investigations.

**Fig 2:**
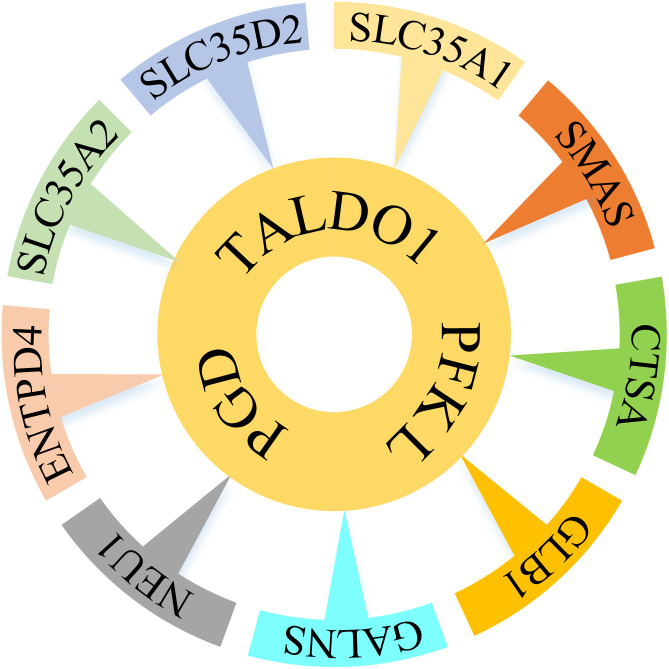
The nine mutual quadruple SL sets among COAD, BRCA, HNSC, UCEC, and STAD. All sets share three genes in common: *PFKL*, *TALDO1*, and.

By increasing the cardinality of the SL sets to four, the number of strictly-selective drug targets increases to nine sets. The genes included in these nine sets are depicted in Fig 2. The characteristics of these genes are discussed in the Discussion section.

**Fig 3:**
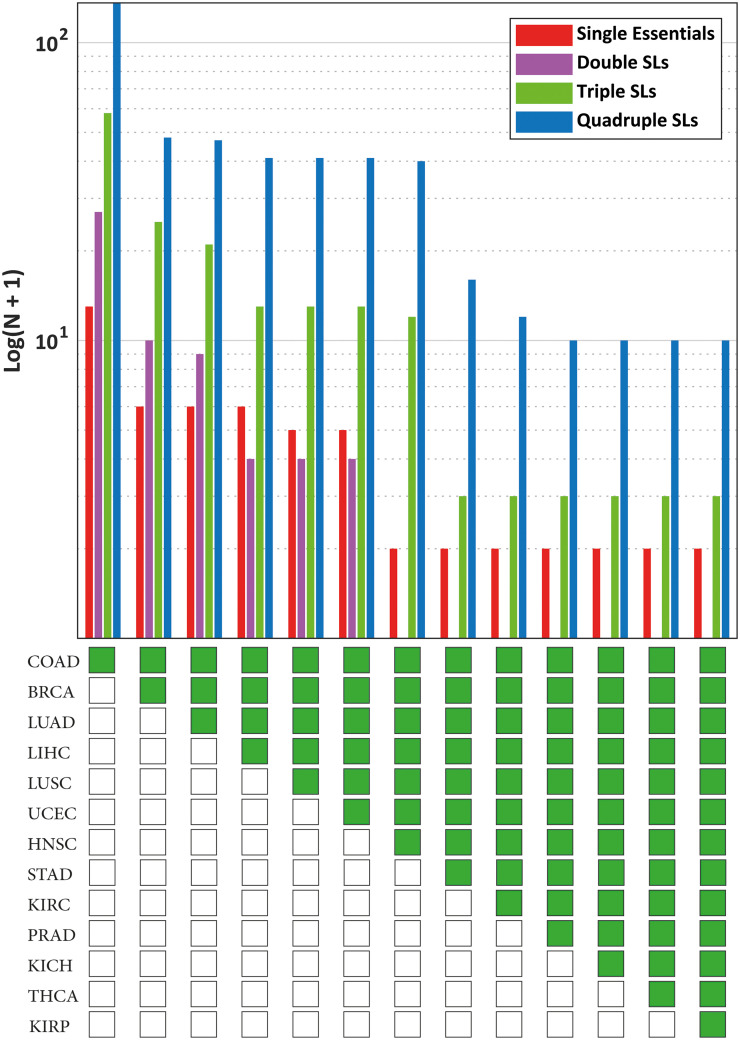
Number of potential selective drug targets identified when different non-tumor tissues were considered protected and COAD was considered the target. The non-tumor tissues considered protected in each column are marked in green below the chart.

To examine how protecting additional non-tumor tissues affects the number of potential solutions, the number of single essential, double SL, triple SL, and quadruple SL solutions that remained selective at each step is presented in Fig 3.

In Fig 3, from left to right, as the number of tissues considered protected increases, the number of selective drug targets decreases. Considering only six non-tumor tissues (including the non-tumor tissue of the colon itself), all double SL sets were disqualified, leaving only one essential gene as a selective target. However, 11 higher-order SL sets (nine quadruple SLs and two triple SLs) remained as *strictly-selective* solutions.

**Fig 4:**
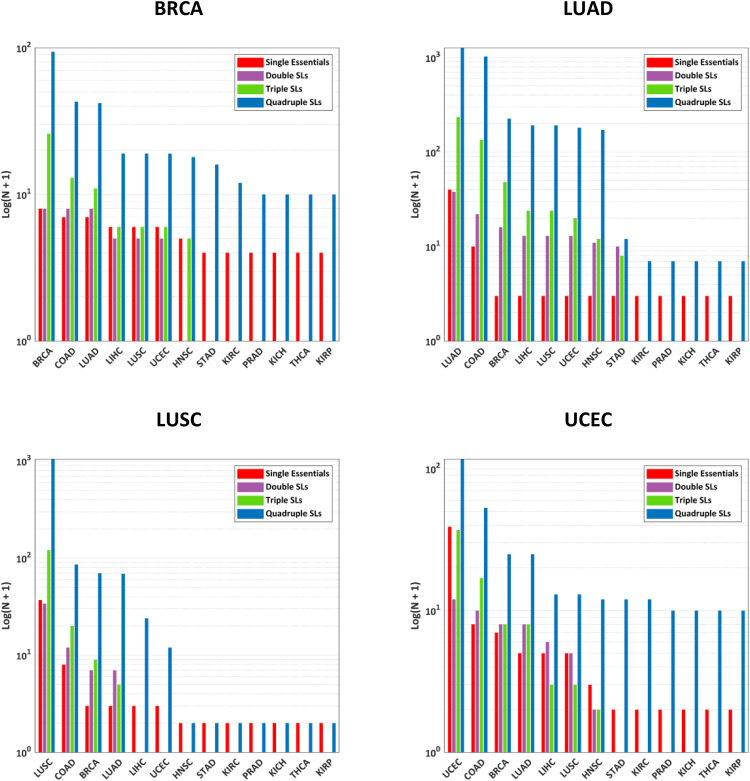
Number of potential selective drug targets identified when different non-tumor tissues were considered protected and BRCA, LUAD, LUSC, and UCEC were considered the targets.

**Fig 5:**
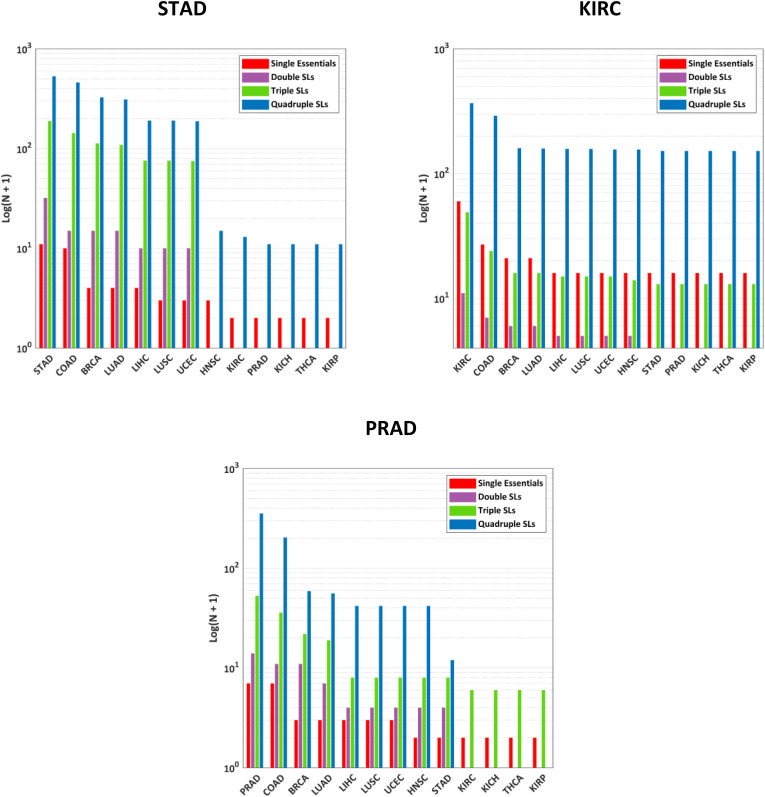
Number of potential selective drug targets identified when different non-tumor tissues were considered protected and STAD, KIRC, and PRAD were considered the targets.

As an example of these higher-order SLs, the simultaneous knockout of *PFKL*, *TALDO1*, *PGD*, and *CMAS* was identified as deleterious for COAD cells, with potentially minimal side effects on the 13 non-tumor tissues. For other cancers, the same figures were generated and reported in Figs 4 and Fig 5.

For these eight cases (shown in Figs 3, 4, 5), no SL set (sets with two targets) was found to meet the *strictly-selectivity* criteria, while higher-order SL sets, especially quadruple SLs, emerged as the dominant source of potential solutions.

### Cases without any essential genes as *strictly-selective* solutions

**Fig 6:**
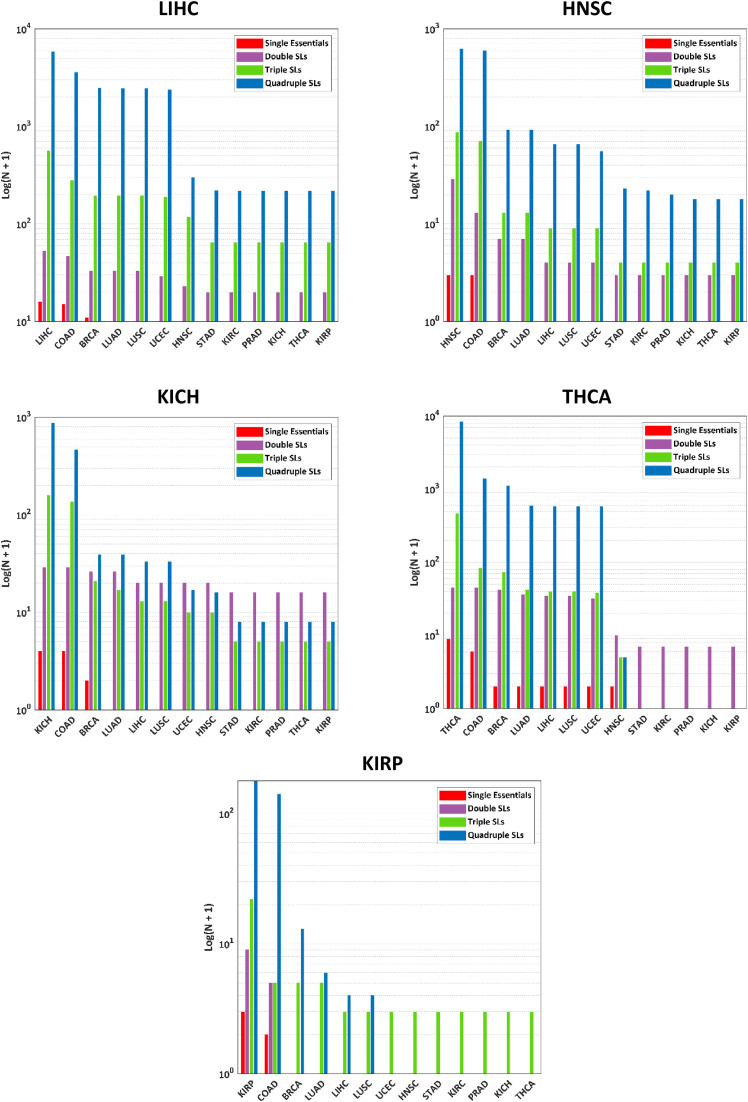
Number of potential selective drug targets identified when different non-tumor tissues were considered protected and LIHC, HNSC, KICH, THCA, and KIRP were considered the targets.

After obtaining *strictly-selective* drug targets for all 13 cancers, it was revealed that no single essential drug target was found for five cases: LIHC, HNSC, KICH, THCA, and KIRP. This suggests that essential genes of these cancers are also essential for at least one non-tumor tissue. Interestingly, for all of these cases, SLs, and more significantly, higher-order SLs, offer effective strategies. The number of selective solutions at each step for these five cases is shown in Fig 6.

### Exploration of the identified solutions across different cancers

**Fig 7:**
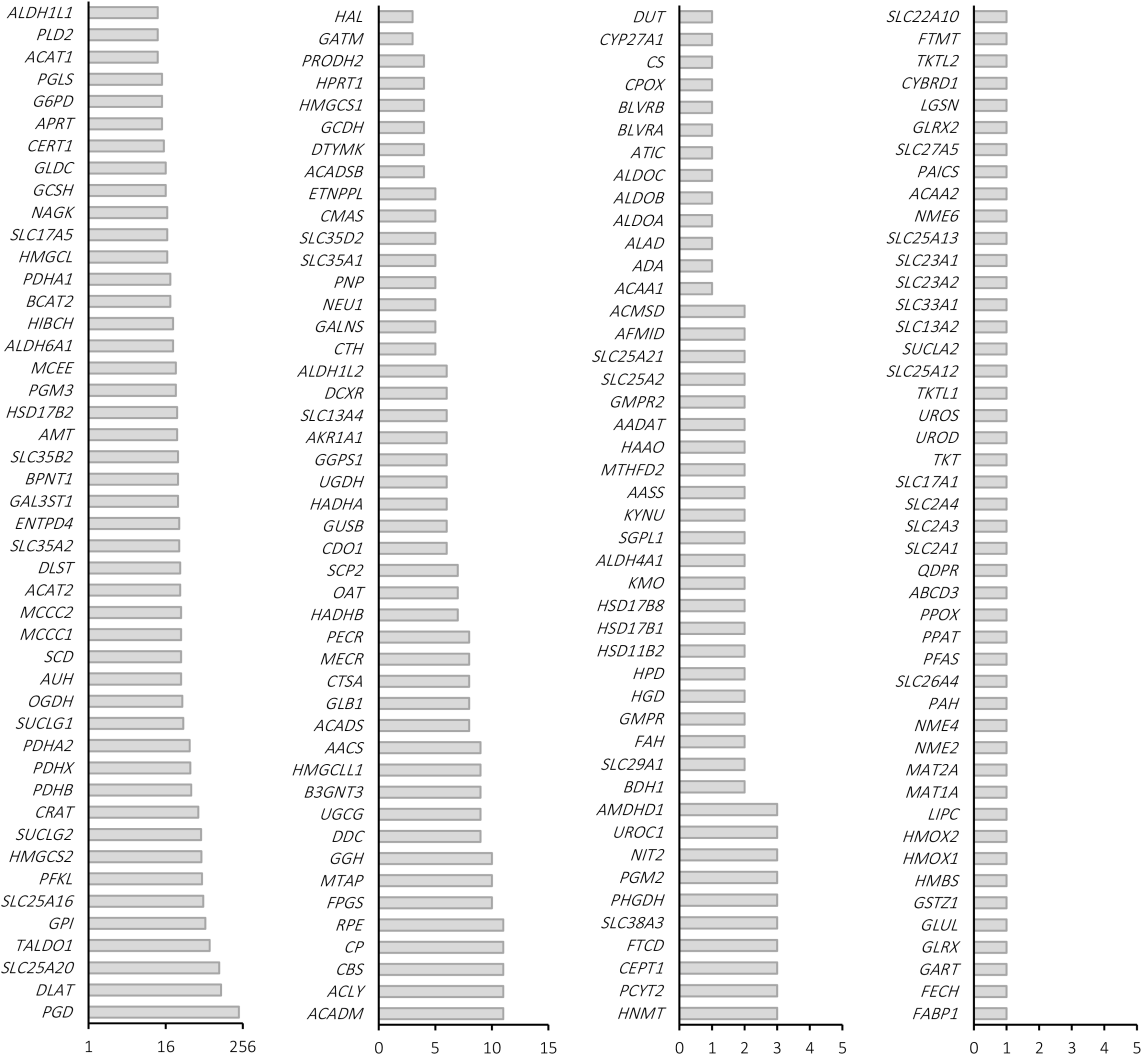
Frequency of different genes across all identified solutions. This figure shows how many times each gene was identified as a target across all single essential and SL solutions.

In this section, different aspects of the obtained solutions are investigated. By considering all the obtained solutions, the most frequently occurring gene, as shown in Fig 7, is Phosphogluconate Dehydrogenase (*PGD*), followed by Dihydrolipoyl transacetylase (*DLAT*) and solute carrier family 25 member 20 (*SLC25A20*). Other important genes, such as Transaldolase 1 (*TALDO1*) and 6-phosphofructokinase liver type (*PFKL*), were targeted in several solutions.

**Fig 8:**
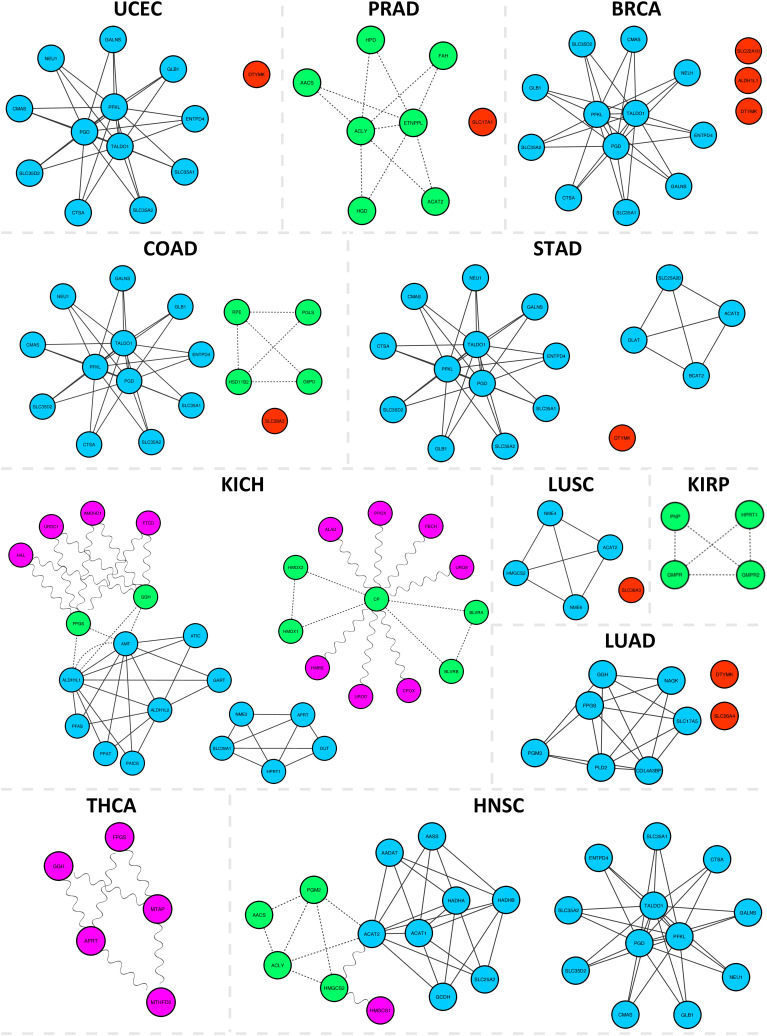
Relationships among genes in the identified *strictly-selective* solutions. Single essential genes are shown in red, double SLs are shown in purple and connected by wavy lines, triple SLs are shown in green and connected by dashed lines, and quadruple SLs are shown in blue and connected by solid lines.

The relationship between different genes in the obtained solutions is visualized in Fig 8 based on their co-occurrence in each SL set. Because various solutions were identified for LIHC and KIRC, their graph is not presented here (see S3 File).

According to Fig 8, COAD, BRCA, HNSC, UCEC, and STAD share a group of nine quadruple SL sets that consist of a core of genes, including *PFKL*, *TALDO1*, and *PGD.* In each set, the fourth gene provides the decisive perturbation and reduces the growth rate of the ΔPFKL,ΔTALDO1,ΔPGD mutant to below 50% of the corresponding cancer cell.

## Discussion

In this work, *strictly-selective* potential drug targets were identified as those that are lethal to cancer cells while remaining inactive not only in the corresponding healthy tissue but also across other non-tumor tissues in the human body. Although for selective targeting of cancer cells, it is a common practice to necessarily target a cancer-specific mutant gene alongside a non-essential gene [39,40], this work did not limit the identification of SL pairs to cancer-specific mutations. Through this unbiased approach, a wider range of solutions was identified, which does include solutions based on the cancer-specific mutations. Note that the provided results are solely based on in silico experiments, and further examinations are crucial for each specific potential case, especially because some of the obtained solutions, such as *SLC38A3*, *RPE*, *HSD11B2*, or *PGLS* identified as a target for different cancers in this study, have been reported as deleterious in mice [58].

To obtain the *strictly-selective* drug targets, 26 context-specific genome-scale metabolic models were reconstructed for non-tumor and cancerous cells of 13 tissues using the rFASTCORMICS algorithm. Afterward, synthetic lethality analysis on the 13 cancerous models was performed using Rapid-SL to identify double, triple, and quadruple SL gene sets, as well as single essential genes. As a result, desired drug targets were identified for each cancer by checking the *strictly-selectivity* criteria on each essential gene or SL gene set using the 13 non-tumor GEMs.

The superiority of higher-order SL sets to provide potential solutions was demonstrated by analyzing the obtained solutions. It also revealed that no *strictly-selective* solution could be provided by single essential genes for LIHC, HNSC, KICH, THCA, and KIRP, and only synthetic and higher-order SL sets were able to offer qualified solutions for these cases. Furthermore, nine quadruple SL sets were identified as *strictly-selective* for COAD, BRCA, HNSC, UCEC, and STAD. These nine sets include three shared genes: *PFKL* (phosphofructokinase), *TALDO1* (transaldolase), and *PGD* (phosphogluconate dehydrogenase), and nine different genes for each set. The three shared genes are among the most frequently identified genes in the solutions and are involved in both glycolysis and the pentose phosphate pathway, both of which are highly implicated in the development of cancers. Due to the crucial role of pentose phosphate pathway overexpression in supporting the anabolic needs and addressing oxidative stress in glycolytic cancer cells [59,60], simultaneously targeting both pathways is expected to be effective against cancers.

Phosphofructokinase is a key enzyme in glycolysis and gluconeogenesis, and its inhibition has been reported to be effective in suppressing colorectal cancer [61]. Additionally, it is known to halt the progression of hepatocellular carcinoma [62] and manage lung cancer [63]. Transaldolase is an enzyme related to the pentose phosphate pathway and has been associated with the development of cancer [64]. This enzyme plays a vital role in the pentose phosphate pathway within fast-dividing cancer cells, serving as a crucial mediator that enables glycolytic cancer cells to meet their anabolic needs and effectively cope with oxidative stress [60]. Additionally, this enzyme is associated with bladder [65], liver [66,67], and breast [68] cancer. The association between higher transaldolase expression and decreased responsiveness to *HER2* inhibitors in breast cancer patients suggests a potential role of transaldolase in cancer drug resistance. Therefore, suppressing transaldolase as a drug target increases the susceptibility of *HER2*-amplified cell lines to *HER2* inhibition [69]. Phosphogluconate dehydrogenase is one of the pentose phosphate pathway enzymes that has recently gained attention due to its crucial role in the development of tumors and maintaining cellular redox balance [70]. Phosphogluconate dehydrogenase is frequently overexpressed in various types of cancer, promoting the ability to grow and spread in different cancer cells, such as breast [71,72], ovarian [73], and lung [74]. Additionally, overexpression of phosphogluconate dehydrogenase is related to the development of resistance to radiotherapy [75] and chemotherapy [70,76,77] in different cancers.

Besides the three shared genes, nine distinct genes exist in each of the nine identified *strictly-selective* potential drug targets. These genes are related to five pathways: *CTSA*, *GLB1*, and *NEU1* are related to the sphingolipid metabolism pathway, which is altered in the development and progression of different cancers, especially colon cancer [78–83]. *SLC35A1*, *SLC35A2*, and *SLC35D2* are related to the solute carrier family involved in Golgi transport. Furthermore, the SLC family has been reported to have a significant role in anticancer drug resistance [84]. *SLC35A2* has been shown to be upregulated in many cancers [85,86], including colon cancer. *CMAS*, or cytidine monophosphate N-acetylneuraminic acid synthetase, is related to amino-sugar metabolism. Knockdown of *CMAS* has anticancer activity in triple-negative breast cancer [87]. *GALNS*, or N-acetylgalactosamine-6-sulfatase, is related to keratan sulfate biosynthesis and is reported to have higher expression in many cancers [88]. *ENTPD4*, or ectonucleoside triphosphate diphosphohydrolase 4, is known to have hydrolase activity and specifically functions as a UDP phosphatase, being related to nucleotide metabolism. However, *ENTPD4* is a novel gene that has been investigated in a few research studies. Recently, a study showed that *ENTPD4* is overexpressed in gastric cancer tissues [89]. Hence, its downregulation or knockout may provide a path for competing cancers.

Most experimentally validated SL interactions reported in the literature involve regulatory or signaling genes [90], reflecting the fact that these areas have been the primary focus of functional genomic screens. In contrast, metabolic synthetic lethality, particularly higher-order combinations involving three or more genes, remains largely unexplored, with only a very limited number of multi-gene SL sets validated experimentally to date. Because existing known SL interactions predominantly involve non-metabolic regulatory genes, they cannot be directly used to validate the metabolic higher-order SL sets identified in this study. This gap reflects a current limitation of the field rather than of our approach, and it underscores the need for future experimental work specifically targeting metabolic gene interactions.

Although our analyses provide systematic predictions of strictly selective metabolic vulnerabilities, several methodological limitations must be acknowledged. The findings rely entirely on genome-scale metabolic models and thus inherit assumptions such as steady-state flux balance and the absence of kinetic and regulatory data (and consequently the associated constraints). Context-specific reconstructions based on transcriptomic data may also introduce inaccuracies, and predictions of higher-order synthetic lethality remain sensitive to model structure and incomplete metabolic knowledge. These limitations emphasize that the predicted SL sets should be viewed as hypotheses requiring experimental validation. Future confirmation of the predicted SSDTs could be achieved using established combinatorial perturbation platforms, including CRISPR/Cas9 gene knockout screens, multiplex CRISPR systems enabling two- to four-gene targeting [91,92], and RNAi-based knockdown approaches such as siRNA or shRNA [93–95]. These perturbation tools can be applied in parallel to matched cancer and non-tumor control cell lines, followed by viability, apoptosis, or clonogenic assays to evaluate both lethality and cancer selectivity. Such validation workflows are commonly used for synthetic lethality testing and represent a practical next step for experimentally confirming the SSDTs, particularly the pan-cancer candidates.

## Conclusion

In this work, for the first time, the superior capability of higher-order SL sets over single essential genes in providing *strictly-selective* drug targets was demonstrated using genome-scale metabolic models. Besides reporting over 500 solutions identified as strictly-selective for 13 types of cancers, nine quadruple SLs are specifically investigated as common solutions between five cancers. Targeting any of these nine SLs results in the disruption of glycolysis and the pentose phosphate pathway along with one of the sphingolipid metabolism, solute carrier family, amino-sugar metabolism, keratan sulfate biosynthesis, or nucleotide metabolism pathways. This simultaneous attack suggests a key to effectively and selectively targeting cancer cells. The provided results are solely based on in-silico experimentation, and further experimental studies are required in future work to examine the effectiveness of the obtained results.

## Supporting information

S1 File An Excel file with 14 sheets of data. “Info” sheet describes the studied cancers by their TCGA name. The other sheets contain the identified strictly-selective genes and gene sets.
(XLSX)

S2 File Detailed information on all genes of potential SSDTs of the colon cancer. (PDF)

S3 File The interaction graphs for LIHC and KIRC strictly-selective targets. (PDF)
