# Supplementary material for: Higher order synthetic lethals are keys to minimize cancer treatment effects on non-tumor cells
Source: PLoS One. 2026 Apr 15;21(4):e0342199. doi: 10.1371/journal.pone.0342199 (PMC13082654; doi:10.1371/journal.pone.0342199)
Supplement: S2 File — (PDF) [file pone.0342199.s002.pdf]

Supplementary information for: Detailed information on all genes of potential SSDTs of the colon cancer

Short title: Cancer treatment using higher order synthetic lethals

Mehdi Dehghan Manshadi, Payam Setoodeh, Amin Ramezani, Amin Reza Rajabzadeh, Habil Zare

Correspondence should be addressed to Habil Zare: zare@uthscsa.edu and Payam Setoodeh: payamst@shirazu.ac.ir

Table of Contents

The List of solutions found for COAD 2
SLC38A3 3
RPE 6
G6PD 9
HSD11B2 18
PGLS 22
PFKL 25
TALDO1 29
PGD 33
SLC35A1 38
CMAS 41
CTSA 44
GLB1 48
GALNS 52
NEU1 55
ENTPD4 58
SLC35A2 61
SLC35D2 64
References 67

The List of solutions found for COAD

Single Lethal	Triple SLs			Quadruple SLs			
SLC38A3	RPE	G6PD	HSD11B2	PFKL	TALDO1	PGD	SLC35A1
	RPE	PGLS	HSD11B2	PFKL	TALDO1	PGD	CMAS
				PFKL	TALDO1	PGD	CTSA
				PFKL	TALDO1	PGD	GLB1
				PFKL	TALDO1	PGD	GALNS
				PFKL	TALDO1	PGD	NEU1
				PFKL	TALDO1	PGD	ENTPD4
				PFKL	TALDO1	PGD	SLC35A2
				PFKL	TALDO1	PGD	SLC35D2

Info	
**NCBI gene ID**	10991
**Official symbol**	SLC38A3
**Uniprot protein names**	Sodium-coupled neutral amino acid transporter 3
**Genecards[1] link**	https://www.genecards.org/cgi-bin/carddisp.pl?gene=SLC38A3
**Gepia[2] Link**	http://gepia.cancer-pku.cn/detail.php?gene=SLC38A3
**Proteinatlas[3] Link**	https://www.proteinatlas.org/ENSG00000188338-SLC38A3/pathology

Drugs from DrugBank

**Name**	**DrugBank ID**	**Type**	**Mechanism**	**Role**
Asparagine	DB00174	Small Molecule	Inhibitor	Traget
Histidine	DB00117	Small Molecule	Unknown	Target
L-Glutamine	DB00130	Small Molecule	Unknown	Transporter

Drugs from Genecards

**Name**	**Group**	**Role**	**Mechanism**
L-Glutamine	Nutra	substrate, Transporter	
Asparagine	Nutra	inhibitor, Target	
D-Alanine	Nutra		Glycine receptor agonist
Histidine	Nutra	Target	

Pathways

Neuroinflammation and glutamatergic signaling

Expression

Median (Tumor)	Median (Normal)	Log2(Fold Change)
0.170	0.210	-0.048

Localization for Gene

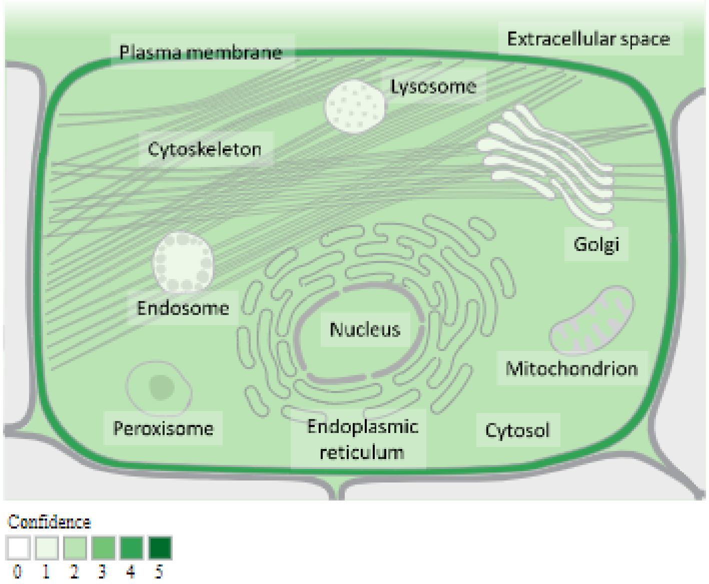

Compartment	Confidence
plasma membrane	4
cytosol	2
endoplasmic reticulum	2
nucleus	2
peroxisome	2
mitochondrion	2
cytoskeleton	2
extracellular	2
golgi apparatus	1
lysosome	1
endosome	1

Expression in different tissues

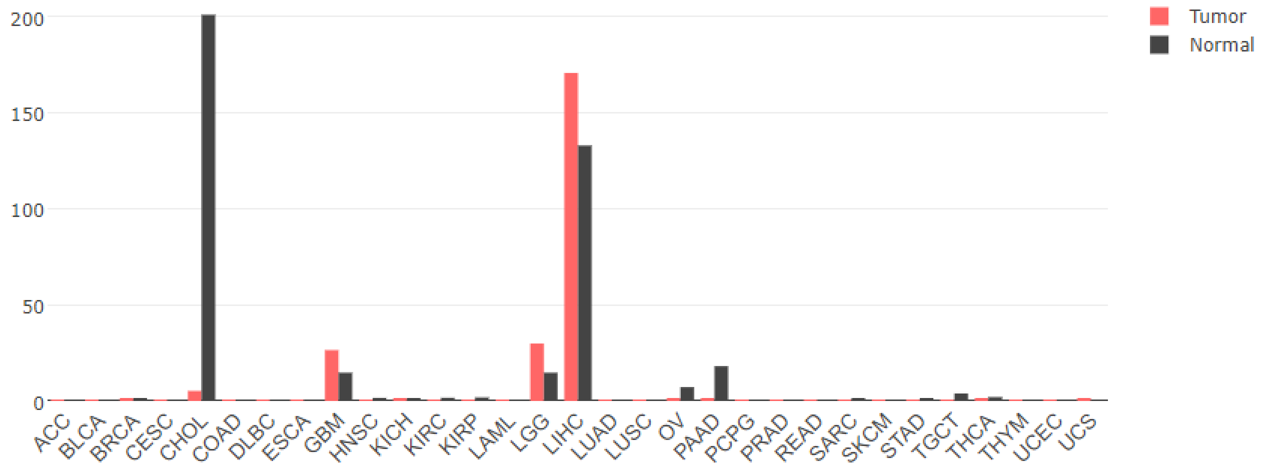

Expression in COAD	Overall Survival

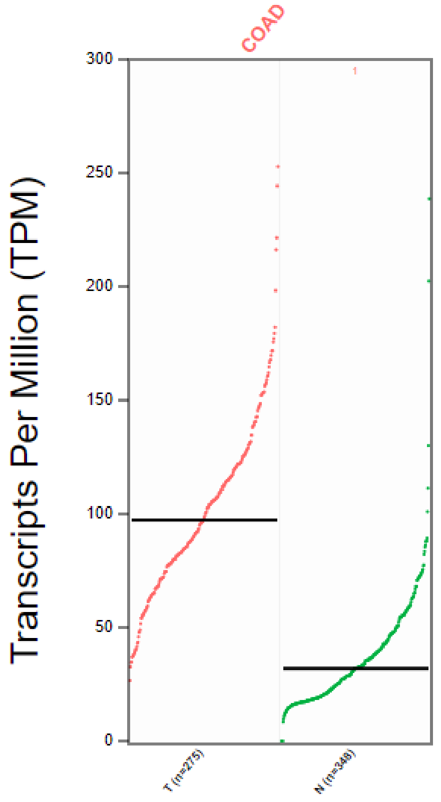

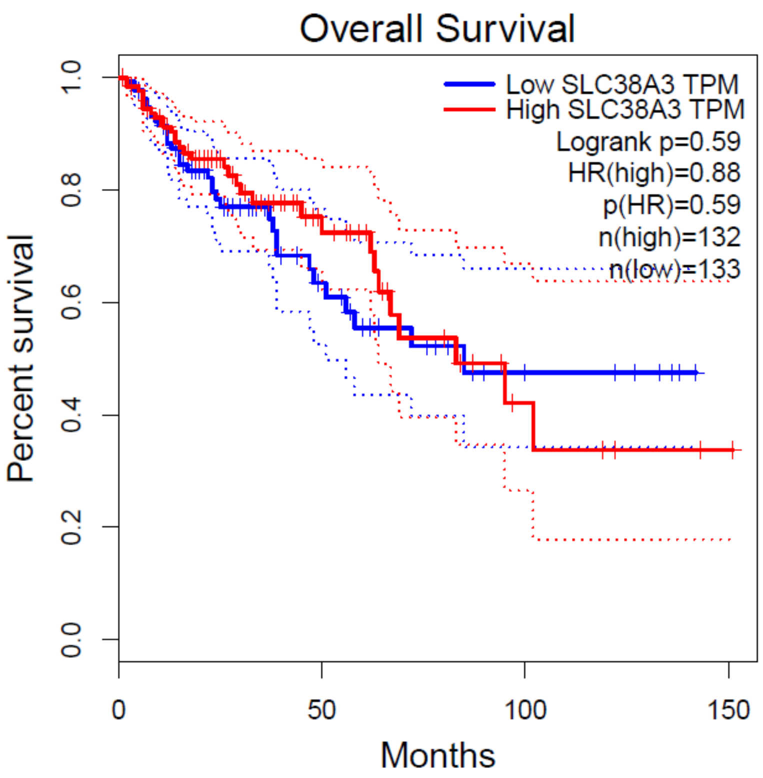

Info	
**NCBI gene ID**	6120
**Official symbol**	RPE
**Uniprot protein names**	Ribulose-phosphate 3-epimerase
**Genecards link**	https://www.genecards.org/cgi-bin/carddisp.pl?gene=RPE&keywords=rpe#localization
**Gepia Link**	http://gepia.cancer-pku.cn/detail.php?gene=RPE
**Proteinatlas Link**	https://www.proteinatlas.org/ENSG00000197713-RPE/pathology

Drugs from DrugBank				
**Name**	**DrugBank ID**	**Type**	**Mechanism**	**Role**

Drugs from Genecards			
**Name**	**Group**	**Role**	**Mechanism**
Ergocalciferol	Nutra	substrate, Enzyme	DNA Polymerase inhibitor, Small Molecule, Antihypocalcemic Agents
Huperzine A	Pharma		NMDA receptor antagonist/AChE inhibitor
Angiotensin II	Pharma		Potent vasopressor and a powerful stimulus for production and release of aldosterone from the adrenal cortex.
Ribose-5-phosphate	Pharma		
Cholinergic Agents	Pharma		DNA Polymerase inhibitor, Small Molecule, Antihypocalcemic Agents

Pathways
Metabolic reprogramming in pancreatic cancer
Pentose phosphate metabolism
Pentose phosphate pathway in senescent cells
Neuroinflammation and glutamatergic signaling

Expression		
Median (Tumor)	Median (Normal)	Log2(Fold Change)
27.94	12.9	1.058

Localization for Gene

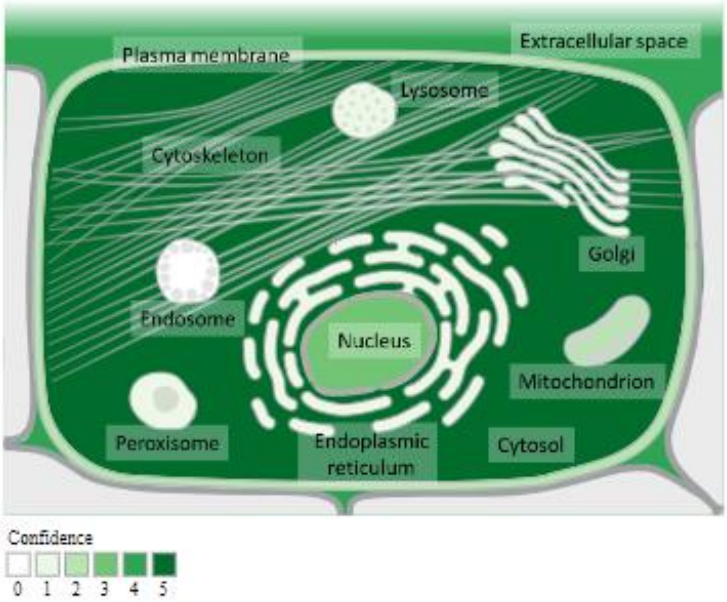

Compartment	Confidence
cytosol	5
extracellular	4
nucleus	3
mitochondrion	2
plasma membrane	2
golgi apparatus	1
lysosome	1
endoplasmic reticulum	1
peroxisome	1
cytoskeleton	1

Expression in different tissues

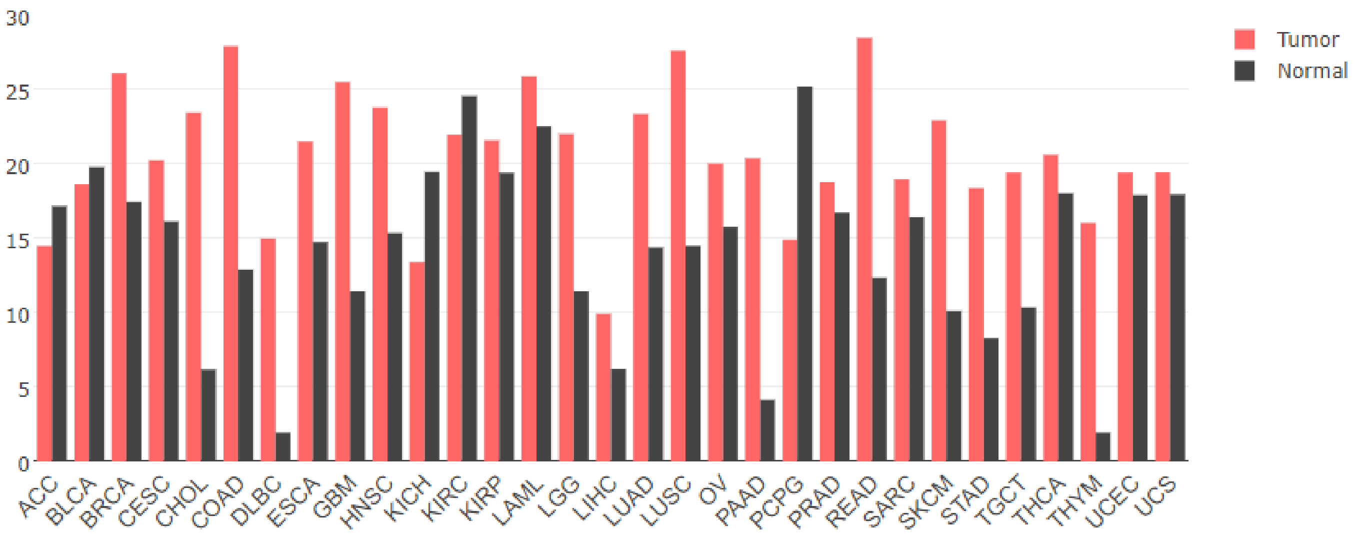

Expression in COAD	Overall Survival

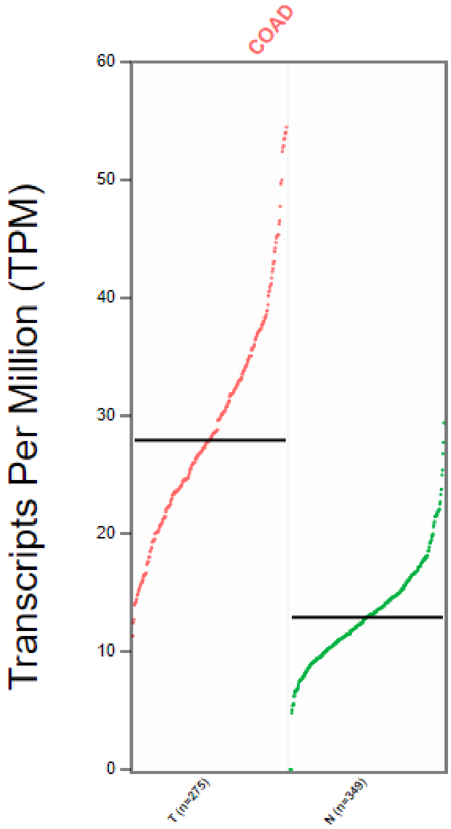

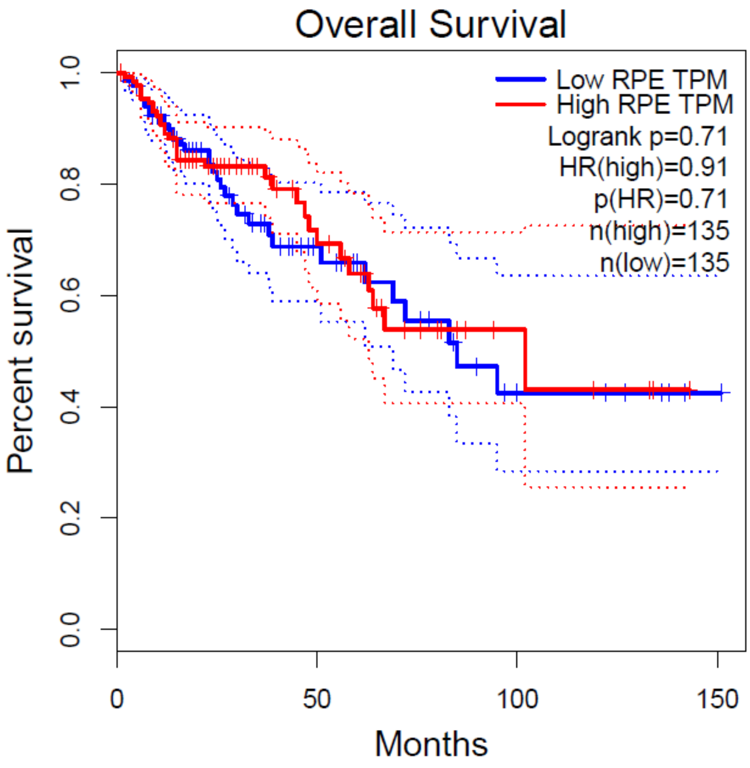

Info	
**NCBI gene ID**	2539
**Official symbol**	G6PD
**Uniprot protein names**	Glucose-6-phosphate 1-dehydrogenase
**Genecards link**	https://www.genecards.org/cgi-bin/carddisp.pl?gene=G6PD&keywords=G6PD#localization
**Gepia Link**	http://gepia.cancer-pku.cn/detail.php?gene=G6pd
**Proteinatlas Link**	https://www.proteinatlas.org/ENSG00000160211-G6PD/pathology

Drugs from DrugBank				
**Name**	**DrugBank ID**	**Type**	**Mechanism**	**Role**
16-Bromoepiandrosterone	DB05107	Small Molecule	Inhibitor	Investigational
Artenimol	DB11638	Small Molecule	Unknown	Approved, Experimental, Investigational
Glycolic acid	DB03085	Small Molecule	Inhibitor	Approved, Investigational
Nicotinamide adenine dinucleotide phosphate	DB03461	Small Molecule	ligand	Experimental

Drugs from Genecards			
**Name**	**Group**	**Role**	**Mechanism**
Chloroquine	Pharma		
Primaquine	Pharma		
Acetylsalicylic acid	Pharma		Cyclooxygenase (COX) inhibitor, Anticoagulants, Anti-Inflammatory Agents, Non-Steroidal
Artesunate	Pharma		derivative of the natural product artemisinin
Ceftriaxone	Pharma		
Dapsone	Pharma		sulfone antibiotic
Doxorubicin	Pharma		Topo II inhibitor,immunosuppresive antineoplastic antibiotic, Small Molecule, Antineoplastic Agents
Glyburide	Pharma		Insulin production modulator, Small Molecule, Hypoglycemic Agents, Kir6 (KATP) channel blocker
Nitrofurantoin	Pharma		
Pioglitazone	Pharma		Small Molecule, Hypoglycemic Agents
Quinidine	Pharma		Anti-Arrhythmia Agents, Small Molecule
Sulfamethoxazole	Pharma		sulfonamide bacteriostatic antibiotic
Tafenoquine	Pharma		

Drugs from Genecards			
**Name**	**Group**	**Role**	**Mechanism**
Methylene blue	Pharma		
Amodiaquine	Pharma		
Chloroprocaine	Pharma		Small Molecule, Anesthetics, Local
Chlorpropamide	Pharma		Oral antihyperglycemic agent, Small Molecule, Hypoglycemic Agents
Ciprofloxacin	Pharma		fluoroquinolone antibiotic
Dabrafenib	Pharma		Inhibitor of BRAF(V600) mutants, Small Molecule, Kinase Inhibitors, RAF Inhibitor
Dimercaprol	Pharma		
Dimercaptosuccinic acid	Pharma		
Flutamide	Pharma		Small Molecule, Antineoplastic Agents, Hormonal
Gliclazide	Pharma		Hypoglycemic Agents, Small Molecule
Glimepiride	Pharma		Sulfonylurea compound, Hypoglycemic Agents, Small Molecule
Glipizide	Pharma		Hypoglycemic Agents, Small Molecule
Hydroxychloroquine	Pharma		Small Molecule
Mafenide	Pharma		
Mepivacaine	Pharma		local anesthetic, Small Molecule, Anesthetics, Local
Metoclopramide	Pharma		Dopamine receptor antagonist, Small Molecule, Antiemetics
Moxifloxacin	Pharma		Fluoroquinolone antibiotic,board specturm, Fluoroquinolone antibiotic
Nicorandil	Pharma		Potassium channel activator, Kir6 (KATP) channel opener and NO donor
Norfloxacin	Pharma		Topoisomerase inhibitor
Ofloxacin	Pharma		Fluoroquinolones,antibiotics
Pegloticase	Pharma		
Perindopril	Pharma		ACE inhibitor, Antihypertensive Agents, Small Molecule, Angiotensin-converting enzyme (ACE) inhibitor
Phenazopyridine	Pharma		
Prilocaine	Pharma		local anesthetic of the amino amide type, Anesthetics, Local, Small Molecule
Probenecid	Pharma		Small Molecule, Uricosuric Agents
Rasburicase	Pharma		
Ropivacaine	Pharma		Small Molecule, Anesthetics, Local
Sulfadiazine	Pharma		Sulfonamide antibiotic

Drugs from Genecards			
**Name**	**Group**	**Role**	**Mechanism**
Sulfanilamide	Pharma		competitive inhibitor for bacterial enzyme dihydropteroate synthetase
Sulfasalazine	Pharma		NF-κB activation inhibitor, Anti-Inflammatory Agents, Non-Steroidal, Antirheumatic Agents, Small Molecule
Sulfisoxazole	Pharma		selective ETA endothelin receptor antagonist, Endothelin ETA antagonist
Tolazamide	Pharma		Small Molecule, Hypoglycemic Agents
Tolbutamide	Pharma		CAMP inhibitor, Small Molecule, Hypoglycemic Agents
Trametinib	Pharma		MEK1 and MEK2 inhibitor, potent and selective, Small Molecule, Kinase Inhibitors
Cisplatin	Pharma		Inhibits DNA synthesis,chemotherapy drug, Potent pro-apoptotic anticancer agent; activates caspase-3
Racepinephrine	Pharma		Bronchodilator Agents, Vasoconstrictor Agents, Small Molecule
Trimethoprim	Pharma		bacteriostatic antibiotic
Glycolic acid	Pharma	inhibitor, Target	
Acetaminophen	Pharma		Small Molecule, Analgesics, Antipyretics
Mefloquine	Pharma		
Pyrimethamine	Pharma		DHFR inhibitor, Potent inhibitor of multidrug and toxin extrusion (MATE) transporters; also DHFR and STAT3 inhibitor
Ascorbic acid	Nutra		antioxidant, water soluble vitamin
Artenimol	Pharma	ligand, Target	
Aminosalicylic acid	Pharma		
Bupivacaine	Pharma		Small Molecule, Anesthetics, Local
Chloramphenicol	Pharma		Inhibits translation(blocking peptidyl transferase)
Meloxicam	Pharma		Nonsteroidal anti-inflammatory drug, Anti-Inflammatory Agents, Non-Steroidal, Small Molecule
Mesalazine	Pharma		Anti-Inflammatory Agents, Non-Steroidal, Small Molecule
Nalidixic acid	Pharma		synthetic quinolone antibiotic
Nitrofural	Pharma		Antibiotic
Nitroglycerin	Pharma		Small Molecule, Antianginal Agents, Vasodilator Agents
Sulfacetamide	Pharma		Sulfonamide antibiotic
Sulfadoxine	Pharma		synthetic analog of para-aminobenzoic acid (PABA)
Sulfamethazine	Pharma		sulfonamide antibacterial
Sulfametopyrazine	Pharma		

Drugs from Genecards			
**Name**	**Group**	**Role**	**Mechanism**
Sodium ascorbate	Nutra		mineral salts of ascorbic acid
Clopidogrel	Pharma		P2Y12 receptor antagonist, Small Molecule, Antithrombotic Agents
Articaine	Pharma		
Busulfan	Pharma		DNA alkylating agent
Carmustine	Pharma		cell-cycle phase nonspecific alkylating antineoplastic agent, Small Molecule, Antineoplastic Agents
Daunorubicin	Pharma		DNA topoisomerase II inhibitor, Small Molecule
Diazepam	Pharma		Small Molecule, Hypnotics And Sedatives
Erythromycin	Pharma		macrolide antibiotic
Esomeprazole	Pharma		H+,K+-ATPase inhibitor, Gi Anti-Ulcer Agents, Proton-Pump Inhibitors, Small Molecule
Lidocaine	Pharma		Anasthetic and class Ib antiarrhythmic agent, Anti-Arrhythmia Agents, Anesthetics, Local, Small Molecule
Nimesulide	Pharma		Non-steroidal anti-inflammatory drug
Oxymetazoline	Pharma		Nasal Decongestants, Small Molecule
Prednisolone	Pharma		Anti-Inflammatory Agents, Glucocorticoids, Small Molecule
Propranolol	Pharma		Small Molecule, Antihypertensive Agents, Anti-Arrhythmia Agents
Tetracaine	Pharma		
Triamcinolone	Pharma		Glucocorticoids, Anti-Inflammatory Agents, Small Molecule
Prasterone	Nutra		Endogenous steroid hormone, Small Molecule
Gluconolactone	Pharma		
Phenol	Pharma		
Pyridoxal phosphate	Nutra		Active form of vitamin B6 serving as a coenzyme for synthesis of amino acids, neurotransmitters (serotonin, norepinephrine), sphingolipids, aminolevulinic acid.
Metabutethamine	Pharma		
Nicotinamide adenine dinucleotide phosphate	Pharma	Target	
Glucose-6-Phosphate	Pharma		
NADPH	Pharma		
Carbaspirin calcium	Pharma		
Furazolidone	Pharma		
Sodium nitrate	Pharma		
Protoporphyrin	Pharma		

Drugs from Genecards			
**Name**	**Group**	**Role**	**Mechanism**
beta-D-glucose 6-phosphate	Pharma		
Chlorproguanil	Pharma		
16-Bromoepiandrosterone	Pharma	Target	
Quinacrine	Pharma		
Ebselen	Pharma		
Sitamaquine	Pharma		
6-Aminonicotinamide	Pharma		6-phosphogluconate dehydrogenase inhibitor
Epiandrosterone	Pharma		
acetaminophen / codeine	Pharma		
acetaminophen / tramadol	Pharma		
amlodipine / atorvastatin / perindopril arginine	Pharma		
antithrombotic agents	Pharma		
articaine / epinephrine	Pharma		
Ascorbic acid (vitamin C), combinations	Pharma		
Ascorbic acid (vitamin C), plain	Pharma		
bisoprolol fumarate / perindopril arginine	Pharma		
erythromycin ethylsuccinate / sulfisoxazole acetyl	Pharma		
lidocaine / prilocaine	Pharma		
lidocaine and tetracaine	Pharma		
moviprep	Pharma		
oxymetazoline and tetracaine	Pharma		
Paracetamol, combinations excl. Psycholeptics (Vicks MediNait Sirup)	Pharma		
sodium nitrite	Pharma		myeloperoxidase inhibitor
sulfamethoxazole / trimethoprim	Pharma		
toluidine blue	Pharma		

Drugs from Genecards			
**Name**	**Group**	**Role**	**Mechanism**
Vitamin B-complex with vitamin C	Pharma		
Vitamin K	Nutra		
Amebicides	Pharma		
Analgesics	Pharma		
Analgesics, Non-Narcotic	Pharma		
Anti-Infective Agents	Pharma		
Anti-Inflammatory Agents	Pharma		
Anti-Inflammatory Agents, Non-Steroidal	Pharma		
Antimalarials	Pharma		
Antioxidants	Pharma		
Antiparasitic Agents	Pharma		
Antiprotozoal Agents	Pharma		
Antipyretics	Pharma		
Antirheumatic Agents	Pharma		
bilirubin	Pharma		
Chloroquine diphosphate	Pharma		Antimalarial drug
Chrysarobin	Pharma		
Cyclooxygenase	Pharma		
Cyclooxygenase Inhibitors	Pharma		
Enzyme Inhibitors	Pharma		
Fibrinolytic Agents	Pharma		
Narcotic Antagonists	Pharma		
Narcotics	Pharma		
Neurotransmitter Agents	Pharma		
Pharmaceutical Solutions	Pharma		
Platelet Aggregation Inhibitors	Pharma		
Purinergic P	Pharma		
Articaine HCl 81	Pharma		dental local anesthetic
CO-TRIMOXAZOLE	Pharma		

Drugs from Genecards			
**Name**	**Group**	**Role**	**Mechanism**
PENICILLIN G POTASSIUM	Pharma		

Pathways
ATM signaling in development and disease
Cori cycle
Glutathione metabolism
Glycolysis in senescence
Metabolic reprogramming in colon cancer
NRF2 pathway
Nuclear receptors meta-pathway
Pentose phosphate metabolism
Pentose phosphate pathway in senescent cells
Sulfation biotransformation reaction
Vitamin D receptor pathway
Vitamin D-sensitive calcium signaling in depression

Expression		
Median (Tumor)	Median (Normal)	Log2(Fold Change)
32.969	15.62	1.031

Localization for Gene

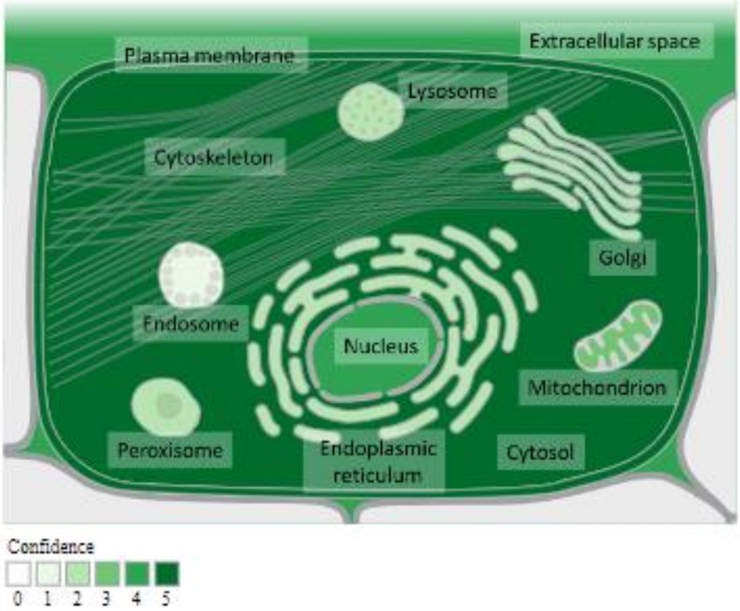

Compartment	Confidence
cytosol	5
cytoskeleton	5
plasma membrane	5
nucleus	4
extracellular	4
mitochondrion	3
golgi apparatus	2
lysosome	2
endoplasmic reticulum	2
peroxisome	2
endosome	1

Expression in different tissues

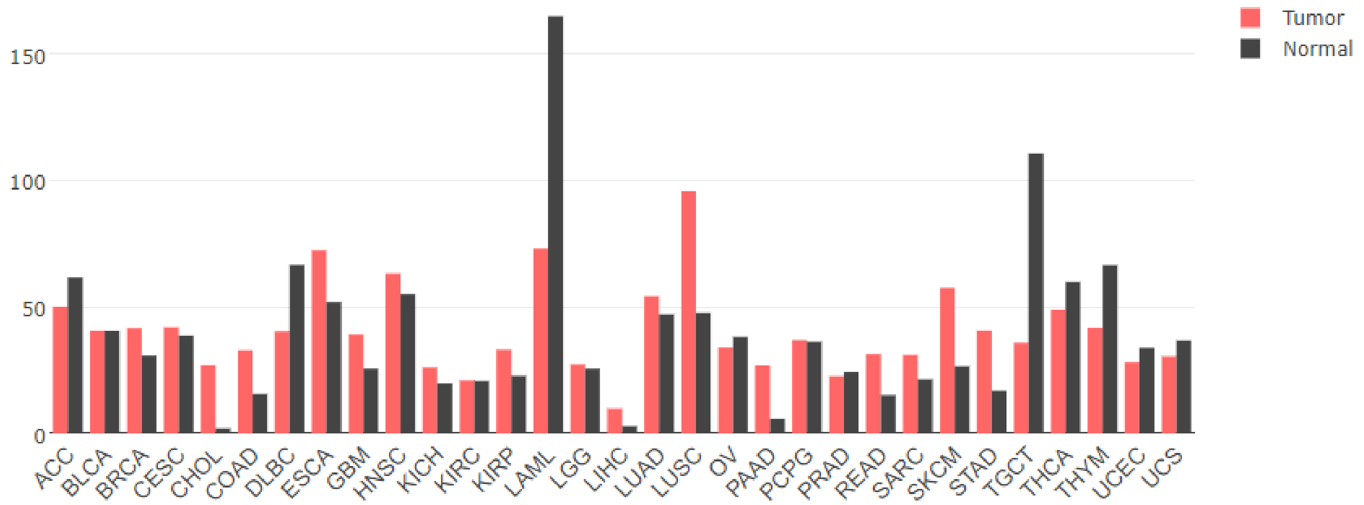

Expression in COAD	Overall Survival

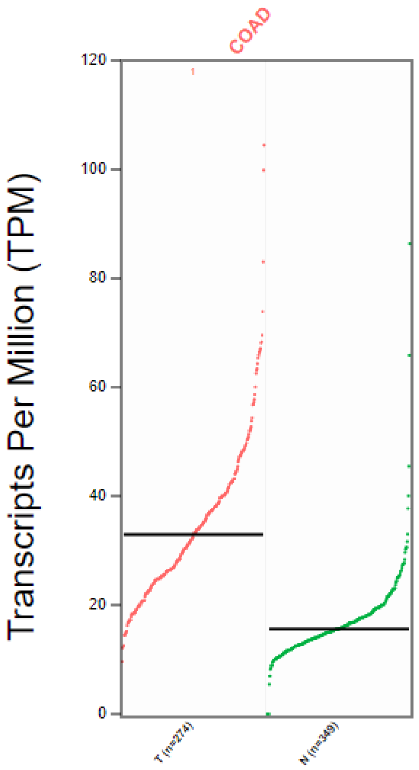

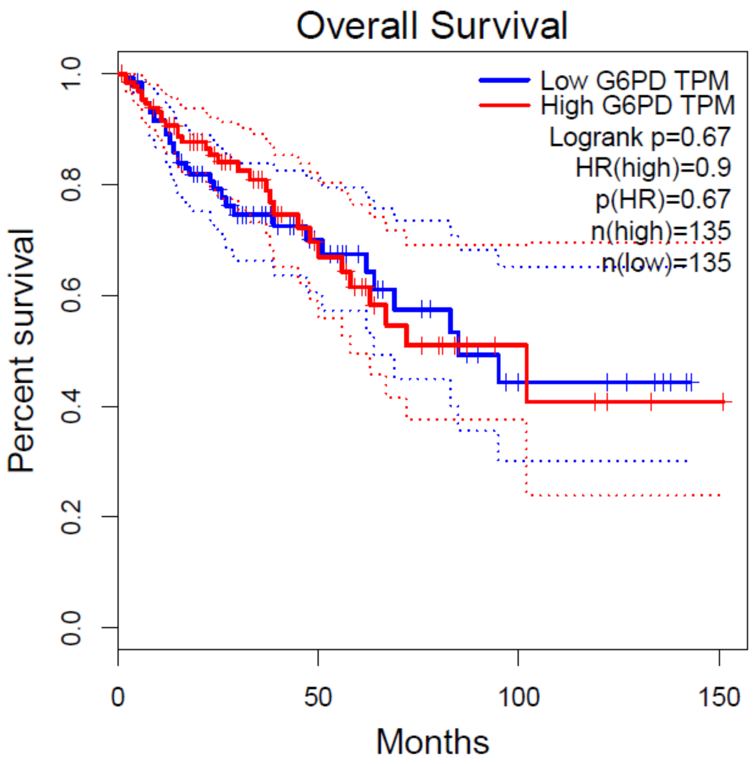

Info	
**NCBI gene ID**	3291
**Official symbol**	HSD11B2
**Uniprot protein names**	11-beta-hydroxysteroid dehydrogenase type 2
**Genecards link**	https://www.genecards.org/cgi-bin/carddisp.pl?gene=HSD11B2&keywords=HSD11B2#localization
**Gepia Link**	http://gepia.cancer-pku.cn/detail.php?gene=HSD11B2
**Proteinatlas Link**	https://www.proteinatlas.org/ENSG00000176387-HSD11B2

Drugs from Web	
**Name**	**Mechanism**
Zearalenone	Zearalenone Inhibits Rat and Human 11β-Hydroxysteroid Dehydrogenase Type 2

Drugs from Genecards			
**Name**	**Group**	**Role**	**Mechanism**
Hydrocortisone	Pharma	substrate, Enzyme	
NADH	Nutra	Target	
Dexamethasone	Pharma	substrate, Enzyme	Glucocorticoids, Anti-Inflammatory Agents, Small Molecule, Anti-inflammatory glucocorticoid
Dexamethasone acetate	Pharma	substrate, Enzyme	
Fludrocortisone	Pharma	substrate, Enzyme	Small Molecule, Anti-Inflammatory Agents
Fluoxymesterone	Pharma	inhibitor, Target	androgen receptor agonist, Anabolic Agents, Antineoplastic Agents, Small Molecule
Hydrocortisone acetate	Pharma	Target	
Hydrocortisone butyrate	Pharma	Target	
Hydrocortisone cypionate	Pharma	Target	
Hydrocortisone phosphate	Pharma	Target	
Hydrocortisone probutate	Pharma	Target	
Hydrocortisone valerate	Pharma	Target	
Methylprednisolone	Pharma	Enzyme	Apoptosis inducer,GR agonist, Anti-Inflammatory Agents, Glucocorticoids, Small Molecule, Glucocorticoid receptor agonist
Prednisolone phosphate	Pharma	substrate, Enzyme	
Phenmetrazine	Pharma		Appetite Depressants, Small Molecule
Corticosterone	Pharma	substrate, Enzyme	
Cortisone	Pharma		
Nadide	Pharma		Coenzyme
Formebolone	Pharma	inhibitor, Target	
Hydrocortisone aceponate	Pharma	Target	

NADPH	Pharma		
Nicotinamide adenine dinucleotide phosphate	Pharma		
Enoxolone	Pharma	Inhibition, Inhibitor	major component of licorice
Carbenoxolone disodium	Pharma		
Adrenosterone	Pharma	substrate, Enzyme	Steroid hormone

Pathways
Glucocorticoid and Mineralcorticoid Metabolism
Glucocorticoid biosynthesis
Oxysterols derived from cholesterol
Prostaglandin synthesis and regulation

Expression		
Median (Tumor)	Median (Normal)	Log2(Fold Change)
68.19	22.461	1.56

Localization for Gene

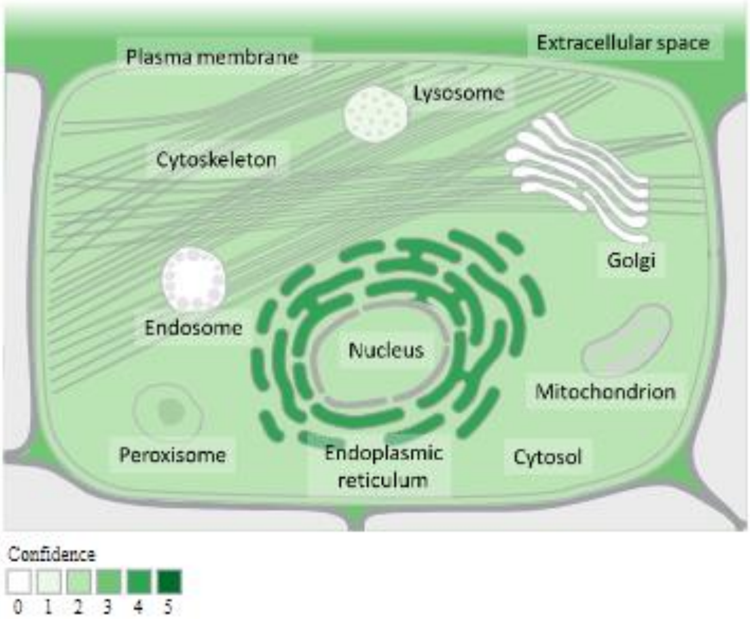

Compartment	Confidence
endoplasmic reticulum	4
extracellular	3
cytosol	2
nucleus	2
peroxisome	2
mitochondrion	2
cytoskeleton	2
plasma membrane	2
lysosome	1
golgi apparatus	0

Expression in different tissues

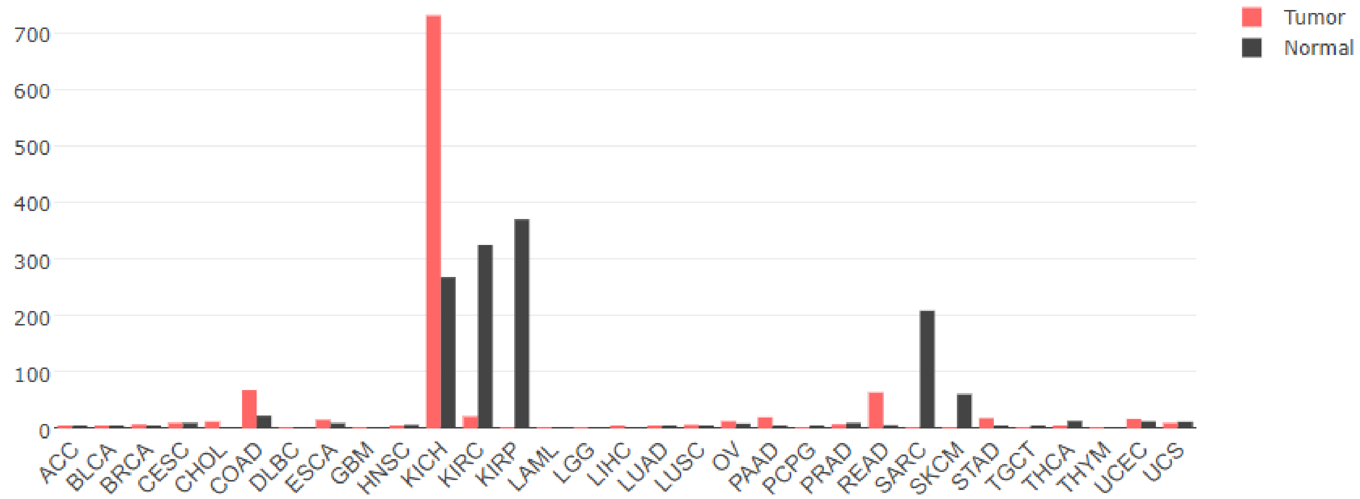

Expression in COAD	Overall Survival

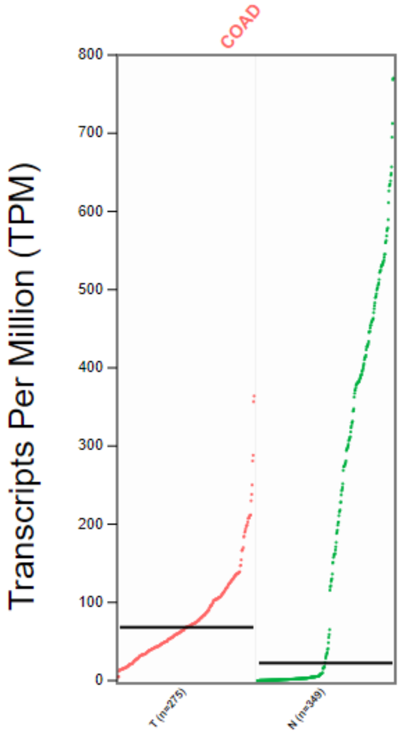

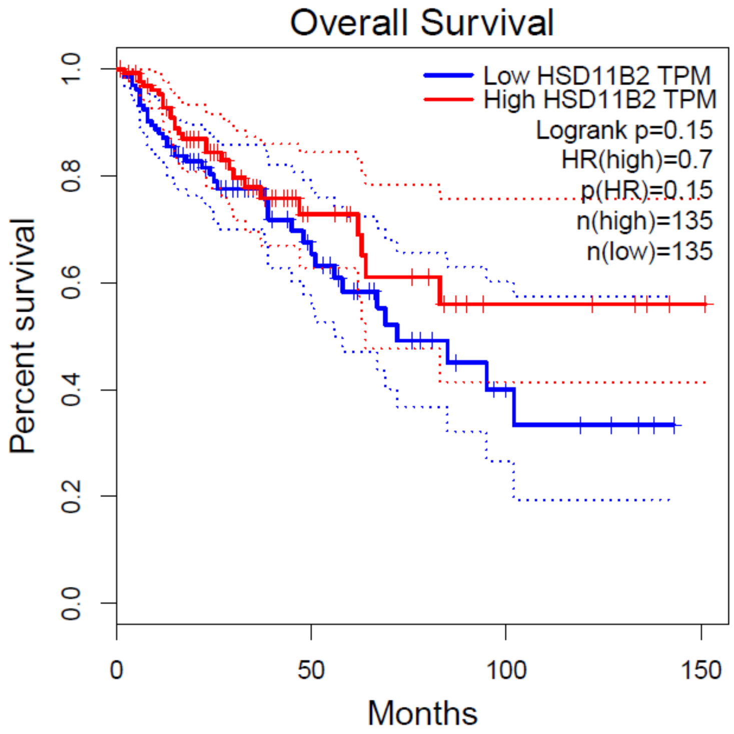

Info	
**NCBI gene ID**	25796
**Official symbol**	PGLS
**Uniprot protein names**	6-phosphogluconolactonase
**Genecards link**	https://www.genecards.org/cgi-bin/carddisp.pl?gene=PGLS&keywords=6-phosphogluconolactonase#localization
**Gepia Link**	http://gepia.cancer-pku.cn/detail.php?gene=PGLS
**Proteinatlas Link**	https://www.proteinatlas.org/ENSG00000130313-PGLS/pathology

Drugs from DrugBank				
**Name**	**DrugBank ID**	**Type**	**Mechanism**	**Role**
Formic acid	DB01942	Small molecule	Unknown	not human pro
Citric acid	DB04272	Small molecule	Unknown	not human pro

Drugs from Genecards			
**Name**	**Group**	**Role**	**Mechanism**
Gluconolactone 24	Pharma		
Magnesium 24	Pharma		
Phenol 24	Pharma		
Water 24	Pharma		
6-phospho-D-gluconic acid 24	Pharma		

Pathways
Pentose phosphate metabolism

Expression		
Median (Tumor)	Median (Normal)	Log2(Fold Change)
80.258	47.130	0.756

Localization for Gene

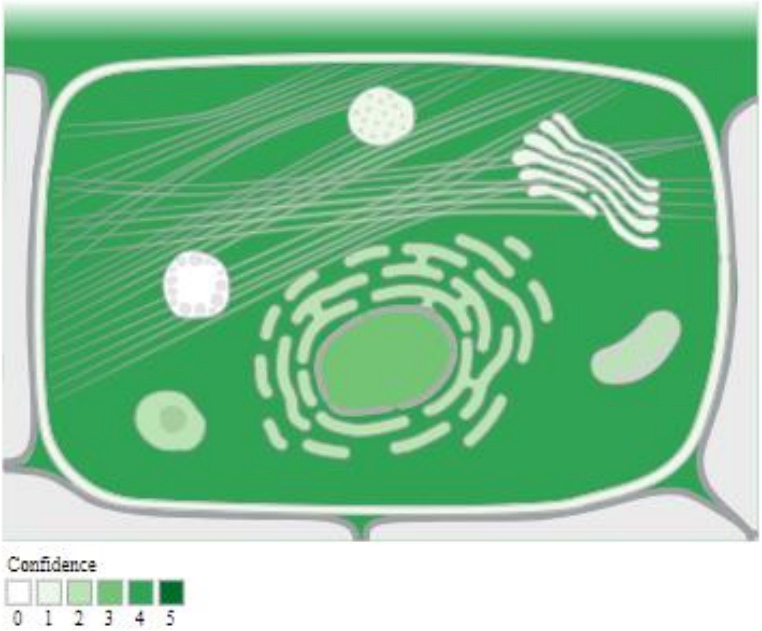

Compartment	Confidence
cytosol	4
extracellular	4
nucleus	3
endoplasmic reticulum	2
peroxisome	2
mitochondrion	2
cytoskeleton	2
golgi apparatus	1
lysosome	1
plasma membrane	1

Expression in different tissues

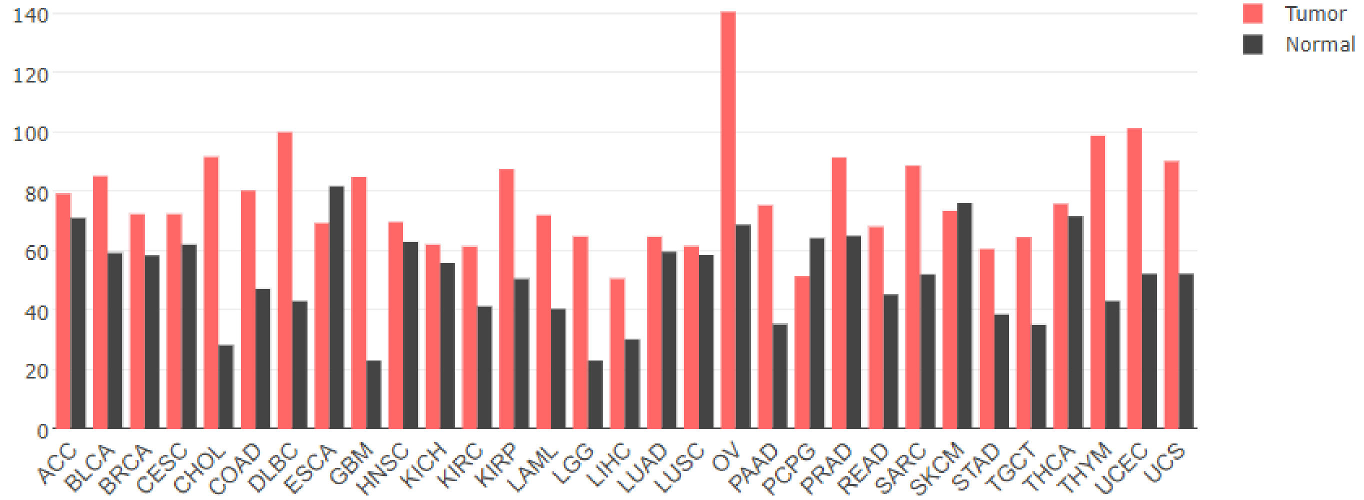

Expression in COAD	Overall Survival

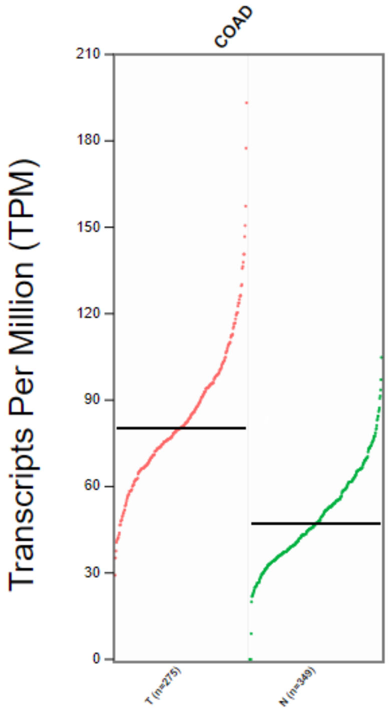

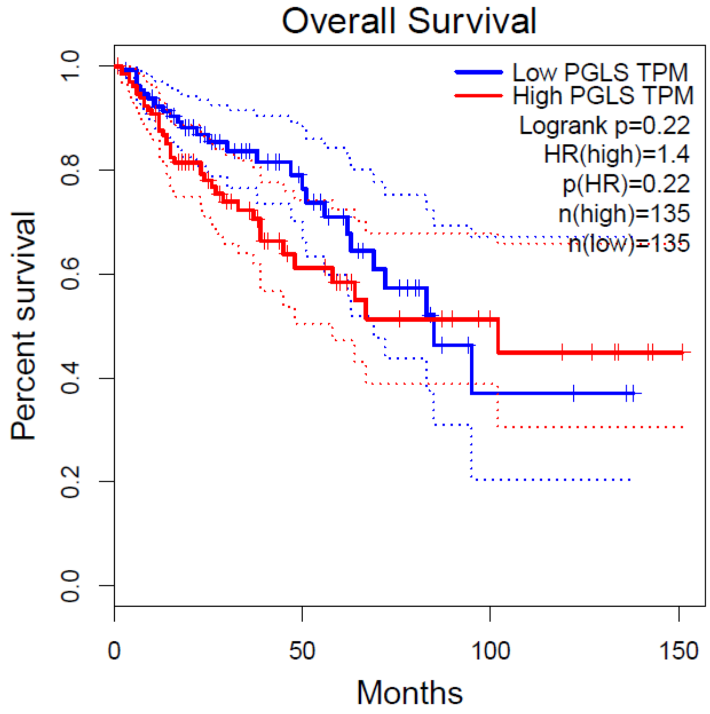

Info	
**NCBI gene ID**	5211
**Official symbol**	PFKL
**Uniprot protein names**	ATP-dependent 6-phosphofructokinase, liver
**Genecards link**	https://www.genecards.org/cgi-bin/carddisp.pl?gene=PFKL&keywords=pfkl#localization
**Gepia Link**	http://gepia.cancer-pku.cn/detail.php?gene=pfkl
**Proteinatlas Link**	https://www.proteinatlas.org/ENSG00000141959-PFKL/pathology

Drugs from DrugBank				
**Name**	**DrugBank ID**	**Type**	**Mechanism**	**Role**
2-Phosphoglycolic Acid	DB02726	Small molecule	Unknown	not human pro
Fructose-6-phosphate	DB04493	Small molecule	Unknown	not human pro

Drugs from Genecards			
**Name**	**Group**	**Role**	**Mechanism**
Magnesium	Pharma		
beta-D-fructofuranose 1,6-bisphosphate	Pharma		
Cytidine-5'-Diphosphate	Pharma		
Cytidine-5'-Triphosphate	Pharma		Pyrimidine nucleoside triphosphate, a substrate in the synthesis of RNA
Fructose-6-phosphate	Pharma		
Guanosine-5'-Diphosphate	Pharma		
Phosphoenolpyruvate	Pharma		
Uridine 5'-triphosphate	Pharma		
Uridine-5'-Diphosphate	Pharma		
Adenosine disphosphate	Pharma		central component of energy storage, metabolism, and signal transduction in vivo; agonist of purinergic receptors
ATP 24	Nutra		
ITP 24	Pharma		

Pathways
Clear cell renal cell carcinoma pathways
Disorders of fructose metabolism
Glycolysis and gluconeogenesis
Insulin signaling
Metabolic reprogramming in colon cancer

Expression		
Median (Tumor)	Median (Normal)	Log2(Fold Change)
118.233	89.958	0.391

Localization for Gene

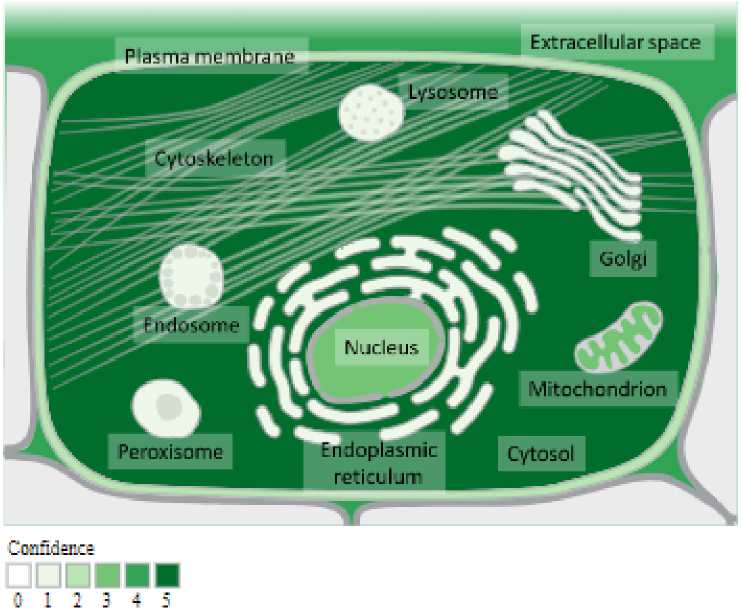

Compartment	Confidence
cytosol	5
extracellular	4
nucleus	3
mitochondrion	3
cytoskeleton	2
plasma membrane	2
golgi apparatus	1
lysosome	1
endosome	1
endoplasmic reticulum	1
peroxisome	1

Expression in different tissues

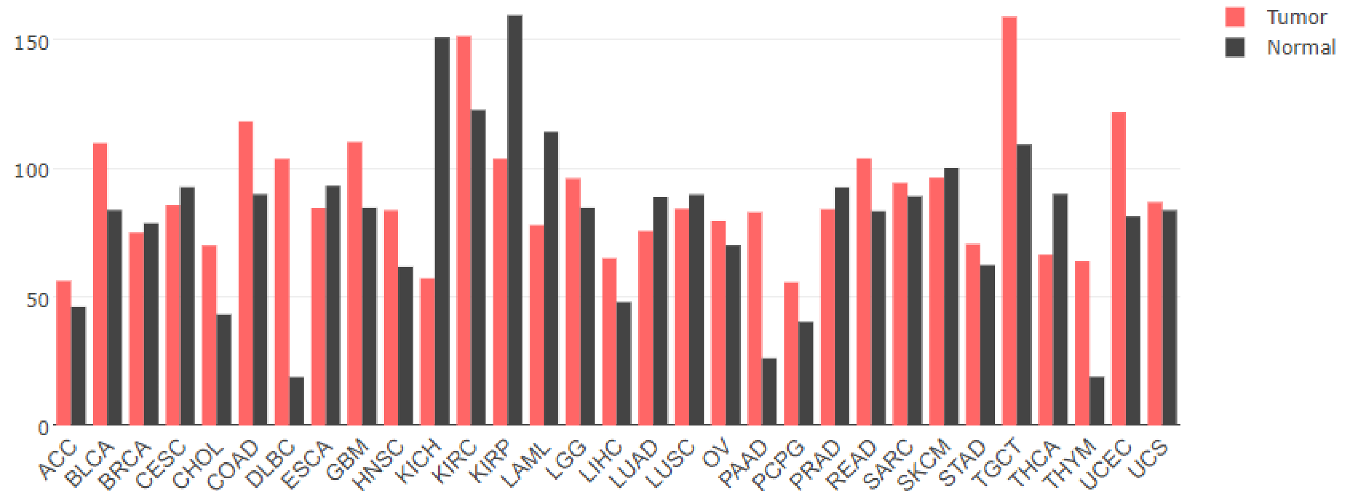

Expression in COAD	Overall Survival
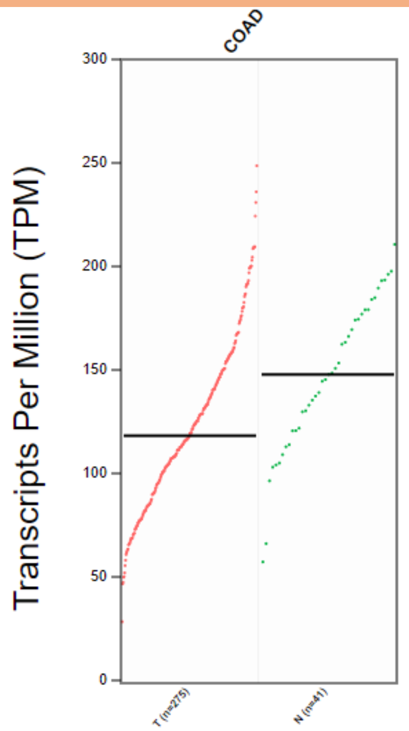	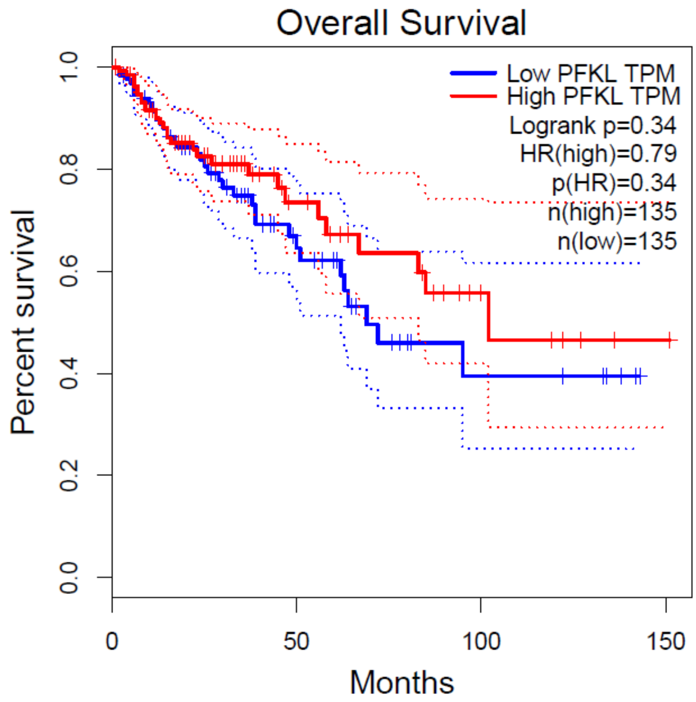

Info	
**NCBI gene ID**	6888
**Official symbol**	TALDO1
**Uniprot protein names**	Transaldolase 1
**Genecards link**	https://www.genecards.org/cgi-bin/carddisp.pl?gene=TALDO1&keywords=TALDO1#localization
**Gepia Link**	http://gepia.cancer-pku.cn/detail.php?gene=TALDO1
**Proteinatlas Link**	https://www.proteinatlas.org/ENSG00000177156-TALDO1/pathology

Drugs from DrugBank				
**Name**	**DrugBank ID**	**Type**	**Mechanism**	**Role**

Drugs from Genecards			
**Name**	**Group**	**Role**	**Mechanism**
Zolpidem	Pharma		
D-glyceraldehyde 3-phosphate	Pharma		
Fructose-6-phosphate	Pharma		
D-erythrose 4-phosphate 24	Pharma		Pyrimidine nucleoside triphosphate, a substrate in the synthesis of RNA

Pathways
Cori cycle
Metabolic reprogramming in colon cancer
Pentose phosphate metabolism

Expression		
Median (Tumor)	Median (Normal)	Log2(Fold Change)
190.929	80.738	1.231

Localization for Gene

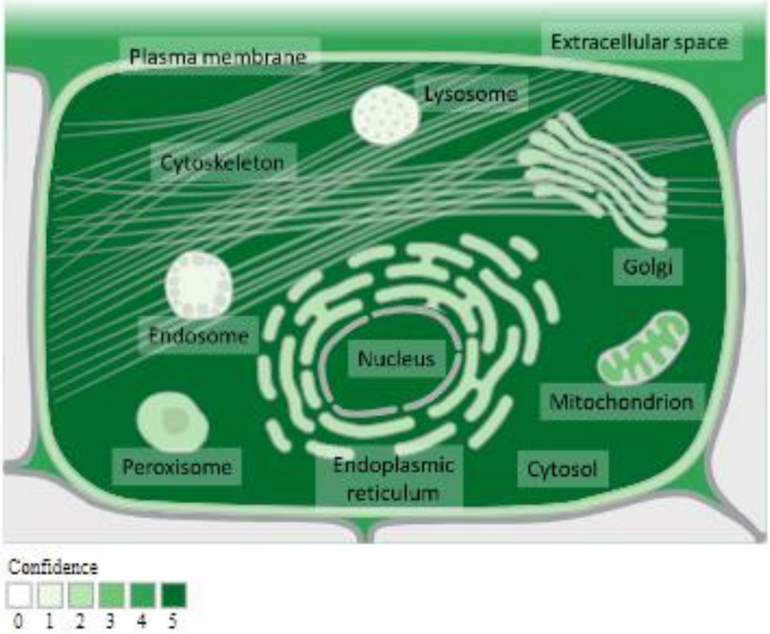

Compartment	Confidence
cytosol	5
nucleus	5
extracellular	4
mitochondrion	3
golgi apparatus	2
endoplasmic reticulum	2
peroxisome	2
cytoskeleton	2
plasma membrane	2
lysosome	1
endosome	1

Expression in different tissues

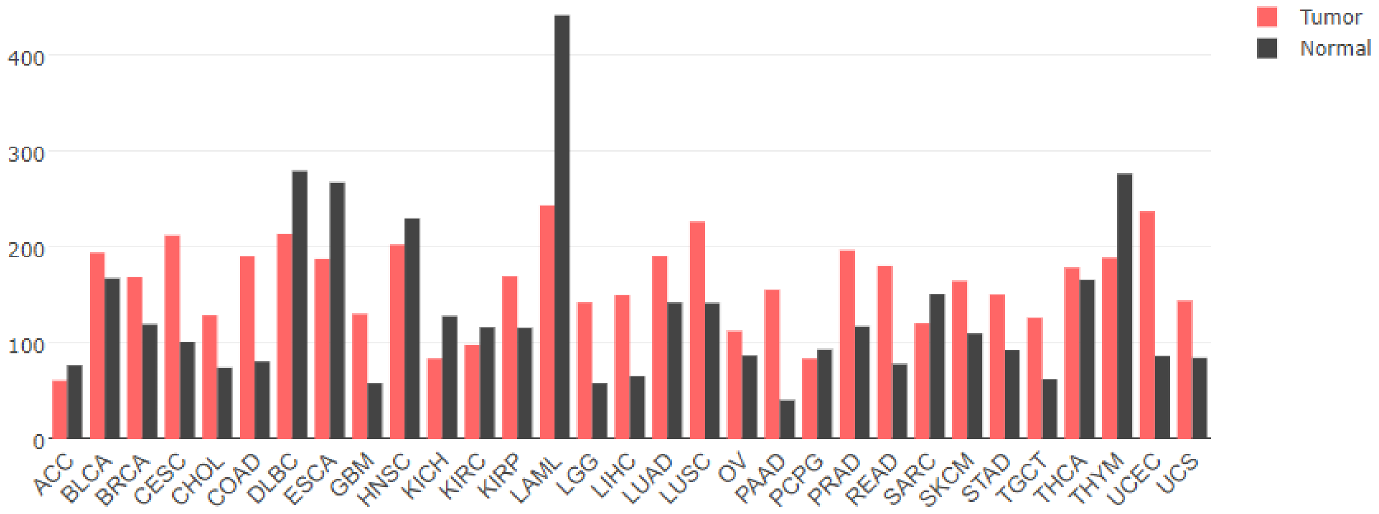

Expression in COAD	Overall Survival

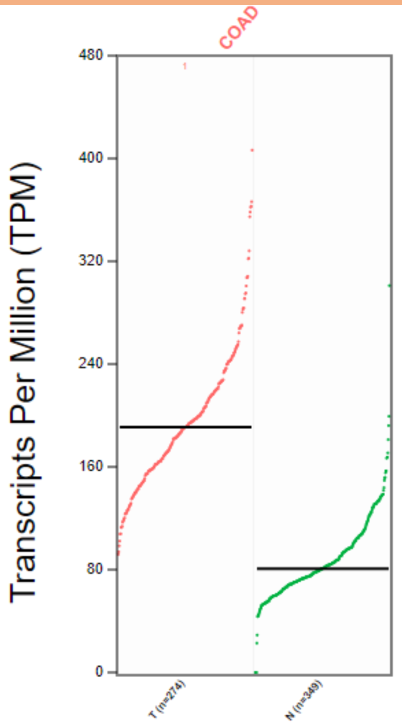

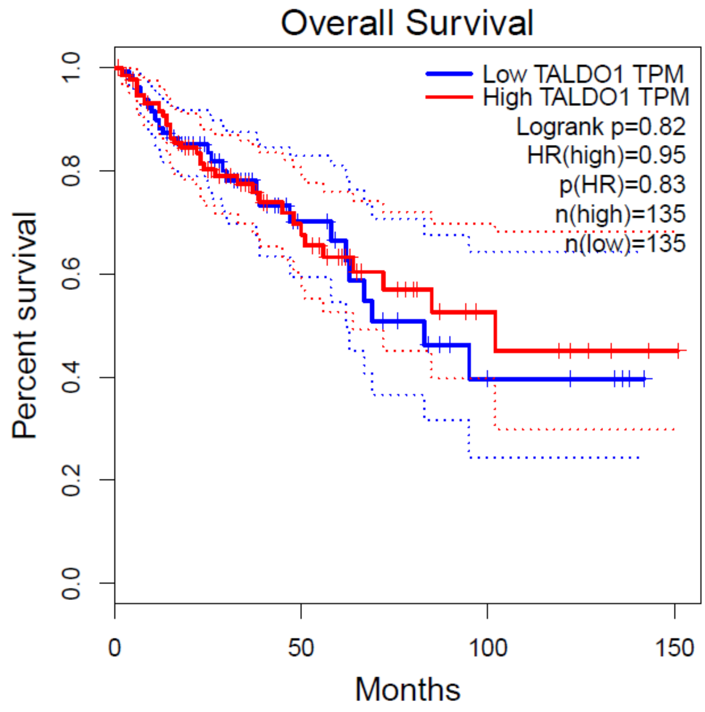

Info	
**NCBI gene ID**	5226
**Official symbol**	PGD
**Uniprot protein names**	phosphogluconate dehydrogenase
**Genecards link**	https://www.genecards.org/cgi-bin/carddisp.pl?gene=PGD&keywords=PGD#localization
**Gepia Link**	http://gepia.cancer-pku.cn/detail.php?gene=PGD
**Proteinatlas Link**	https://www.proteinatlas.org/ENSG00000142657-PGD/pathology

Drugs from DrugBank				
**Name**	**DrugBank ID**	**Type**	**Mechanism**	**Role**
**6-phospho-D-gluconic acid**	DB02076		unknown	
**Nicotinamide 8-bromo-adenine dinucleotide phosphate**	DB03962		unknown	
**Dacarbazine**	DB00851		inhibitor	
**Gadopentetic acid**	DB00789		inhibitor	
**Ketotifen**	DB00920		inhibitor	
**Ritodrine**	DB00867		inhibitor	
**Meloxicam**	DB00814		inhibitor	
**Furosemide**	DB00695		inhibitor	
**Methotrexate**	DB00563		inhibitor	

Drugs from Genecards			
**Name**	**Group**	**Role**	**Mechanism**
Dacarbazine	Pharma	inhibitor, Target	Antineoplastic(malignant melanoma and sarcomas), Small Molecule, Antineoplastic Agents
Furosemide	Pharma	inhibitor, Enzyme	GABAA receptors antagonist, Small Molecule, Diuretics, Antihypertensive Agents, Na+/2Cl-/K+ (NKCC) symporter inhibitor. Also antagonizes GABAA
Ketotifen	Pharma	inhibitor, Target	Anti-Allergic Agents, Small Molecule
Meloxicam	Pharma	inhibitor, Enzyme	Nonsteroidal anti-inflammatory drug, Anti-Inflammatory Agents, Non-Steroidal, Small Molecule
Methotrexate	Pharma	inhibitor, Enzyme	Folate antagonist,inhibits DFHR, Small Molecule
Ritodrine	Pharma	inhibitor, Enzyme	Tocolytic Agents, Small Molecule
Gadopentetic acid	Pharma	inhibitor, Target	

Buserelin	Pharma		
Cetrorelix	Pharma		gonadotropin-releasing hormone (GnRH) antagonist, Peptide, Fertility Agents
Estradiol	Pharma		Sex hormone, Small Molecule, Hormone Replacement Agents, Endogenous ER agonist
Follitropin	Pharma		Protein, Fertility Agents
Ganirelix	Pharma		
Polyestradiol phosphate	Pharma		
Progesterone	Pharma		Contraceptives, Small Molecule, Endogenous progesterone receptor agonist
Licorice	Nutra		
Penicillamine	Pharma		
Phenobarbital	Pharma		Anticonvulsants, Hypnotics And Sedatives, Small Molecule
Angiotensin II	Pharma		Potent vasopressor and a powerful stimulus for production and release of aldosterone from the adrenal cortex.
Carbon dioxide	Pharma		
Phenol	Pharma		
Manganese	Nutra		
6-phospho-D-gluconic acid	Pharma		
NADPH	Pharma		
Nicotinamide 8-bromo-adenine dinucleotide phosphate	Pharma		
Nicotinamide adenine dinucleotide phosphate	Pharma		
Ribose-5-phosphate	Pharma		
Antipsychotic Agents	Pharma		
Chorionic Gonadotropin	Pharma		
Estradiol 17 beta-cypionate	Pharma		
Estradiol 3-benzoate	Pharma		
Hormone Antagonists	Pharma		
Hormones	Pharma		
Pharmaceutical Solutions	Pharma		
Progestins	Pharma		
Prolactin Release-Inhibiting Factors	Pharma		Antineoplastic(malignant melanoma and sarcomas), Small Molecule, Antineoplastic Agents

Pathways
Metabolic reprogramming in colon cancer
NRF2 pathway
Nuclear receptors meta-pathway
Pentose phosphate metabolism

VEGFA-VEGFR2 signaling

Expression		
Median (Tumor)	Median (Normal)	Log2(Fold Change)
97.288	31.899	1.579

Localization for Gene

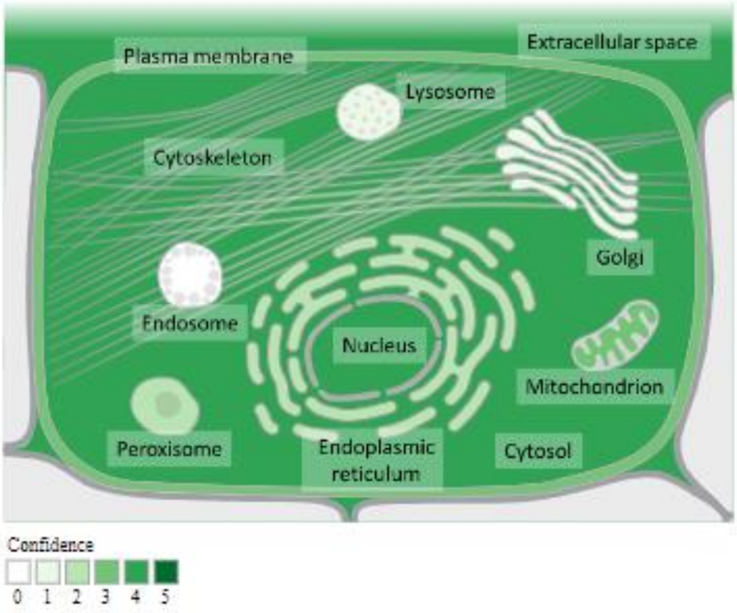

Compartment	Confidence
cytosol	4
nucleus	4
extracellular	4
mitochondrion	3
plasma membrane	3
endoplasmic reticulum	2
peroxisome	2
cytoskeleton	2
golgi apparatus	1
lysosome	1

Expression in different tissues

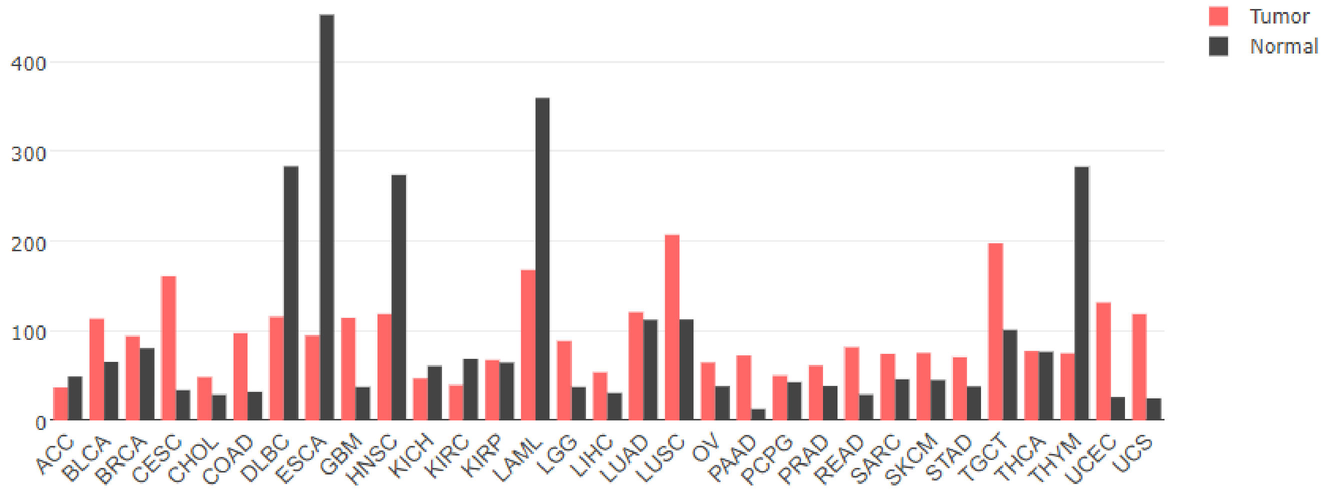

Expression in COAD	Overall Survival

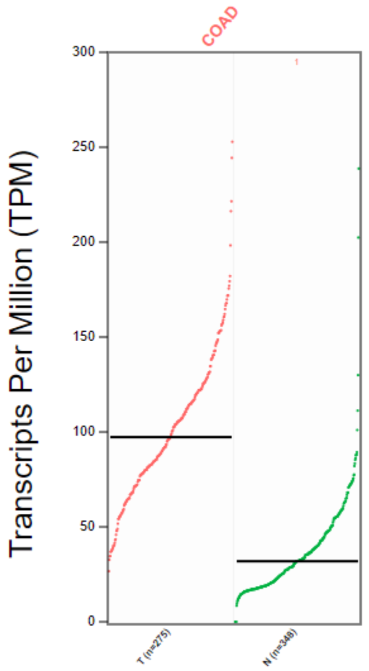

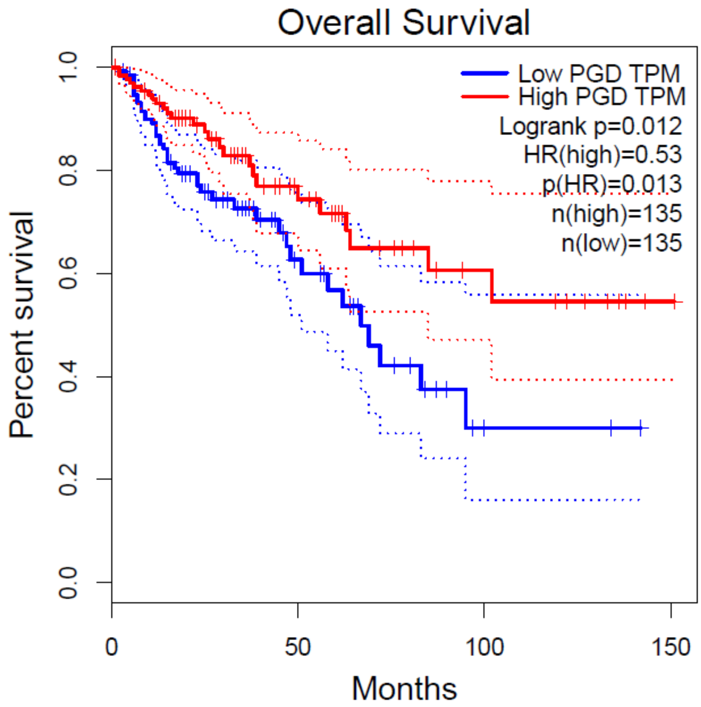

Info	
**NCBI gene ID**	10559
**Official symbol**	SLC35A1
**Uniprot protein names**	solute carrier family 35 member A1
**Genecards link**	https://www.genecards.org/cgi-bin/carddisp.pl?gene=SLC35A1&keywords=SLC35A1#aliases_descriptions
**Gepia Link**	http://gepia.cancer-pku.cn/detail.php?gene=SLC35A1
**Proteinatlas Link**	https://www.proteinatlas.org/ENSG00000164414-SLC35A1/pathology

Drugs from DrugBank				
**Name**	**DrugBank ID**	**Type**	**Mechanism**	**Role**

Drugs from Genecards			
**Name**	**Group**	**Role**	**Mechanism**

Pathways from Reactome
Asparagine N-linked glycosylation
Biosynthesis of the N-glycan precursor (dolichol lipid-linked oligosaccharide, LLO) and transfer to a nascent protein
Defective SLC35A1 causes congenital disorder of glycosylation 2F (CDG2F)
Defective SLC35A1 causes congenital disorder of glycosylation 2F (CDG2F)
Disease
Disorders of transmembrane transporters
Metabolism of proteins
Post-translational protein modification
Sialic acid metabolism
SLC transporter disorders
SLC-mediated transmembrane transport
Synthesis of substrates in N-glycan biosythesis
Transport of nucleotide sugars
Transport of small molecules
Transport of vitamins, nucleosides, and related molecules

Expression		
Median (Tumor)	Median (Normal)	Log2(Fold Change)
?	?	?

Localization for Gene

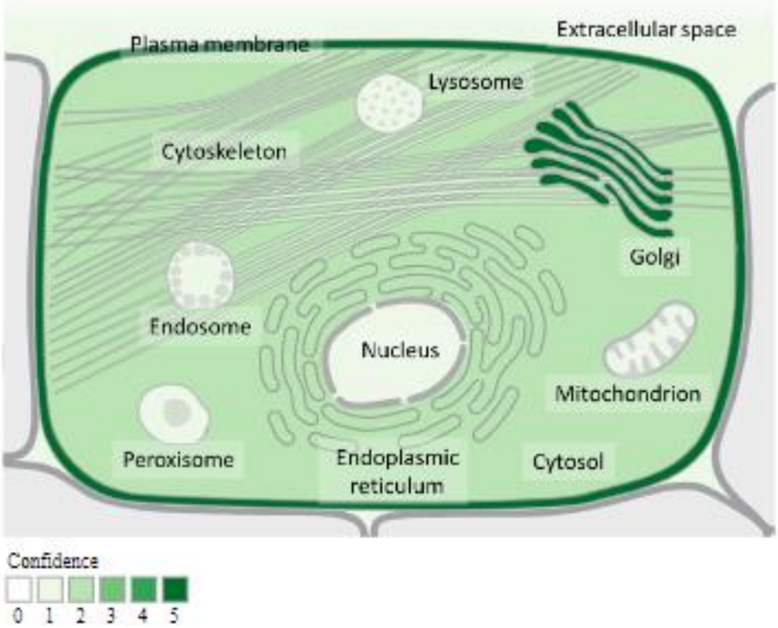

Compartment	Confidence
golgi apparatus	5
plasma membrane	5
cytosol	2
endoplasmic reticulum	2
lysosome	1
endosome	1
nucleus	1
peroxisome	1
mitochondrion	1
cytoskeleton	1
extracellular	1

Expression in different tissues

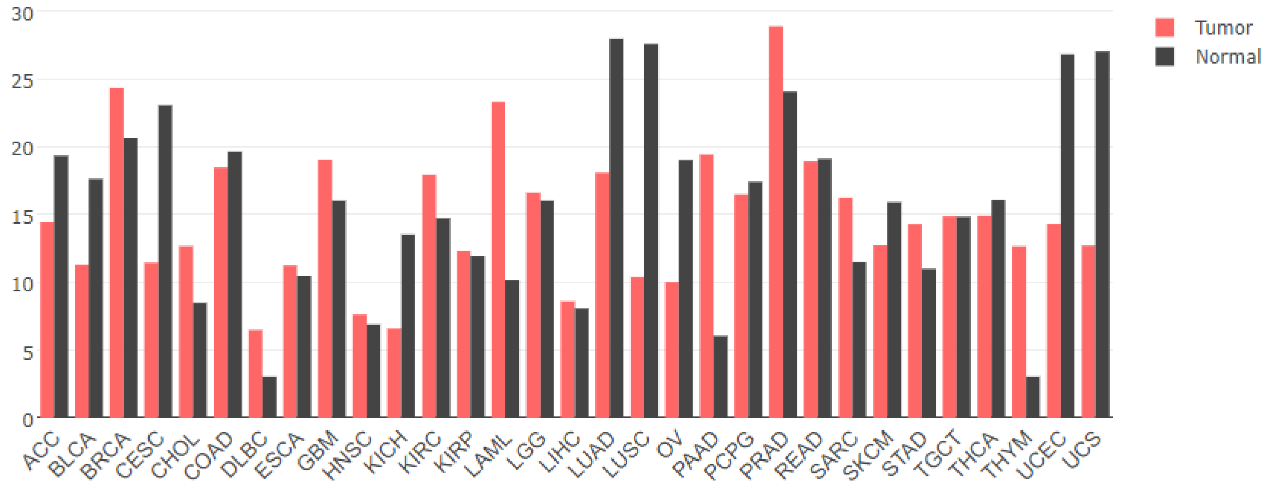

Expression in COAD	Overall Survival

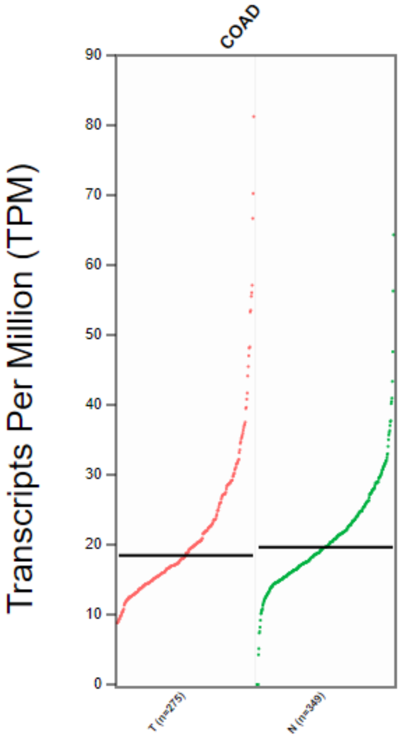

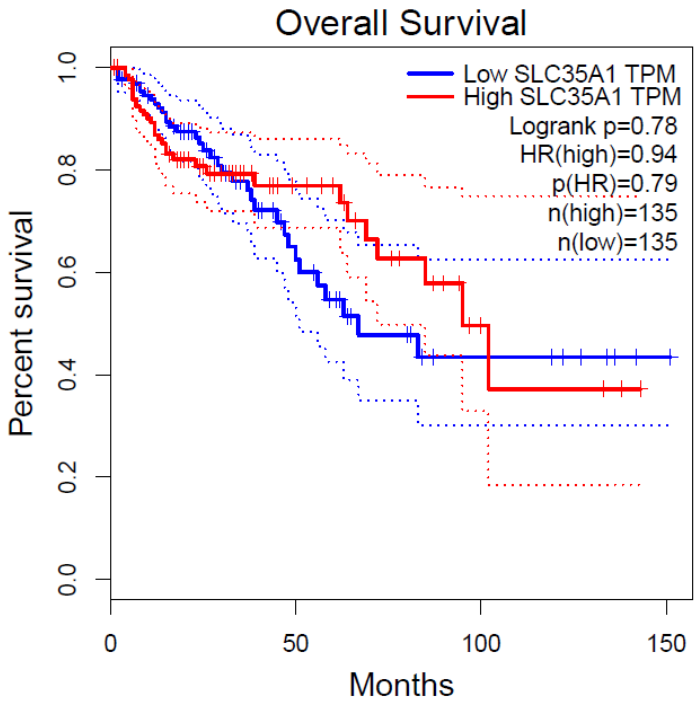

Info	
**NCBI gene ID**	55907
**Official symbol**	CMAS
**Uniprot protein names**	cytidine monophosphate N-acetylneuraminic acid synthetase
**Genecards link**	https://www.genecards.org/cgi-bin/carddisp.pl?gene=CMAS
**Gepia Link**	http://gepia.cancer-pku.cn/detail.php?gene=CMAS
**Proteinatlas Link**	https://www.proteinatlas.org/ENSG00000111726-CMAS/pathology

Drugs from DrugBank

**Name**	**DrugBank ID**	**Type**	**Mechanism**	**Role**
Cytidine-5'-Monophosphate-5-N-Acetylneuraminic Acid	DB02485		unknown	
Cytidine-5'-Diphosphate	DB04555		unknown	not human

Drugs from Genecards

**Name**	**Group**	**Role**	**Mechanism**
Phosphoric acid	Pharma		
Pyrophosphoric acid	Pharma		
Cytidine-5'-Monophosphate-5-N-Acetylneuraminic Acid	Pharma	Target	
Cytidine-5'-Triphosphate	Pharma		Pyrimidine nucleoside triphosphate, a substrate in the synthesis of RNA
N-Carbamoylaspartic acid	Pharma		An intermediary product in pyrimidine biosynthesis.
Aceneuramic acid	Pharma		Human Endogenous Metabolite
Cytidine-5'-Monophosphate	Pharma		

Pathways from Reactome
Asparagine N-linked glycosylation
Biosynthesis of the N-glycan precursor (dolichol lipid-linked oligosaccharide, LLO) and transfer to a nascent protein
Metabolism of proteins
Post-translational protein modification
Sialic acid metabolism
Synthesis of substrates in N-glycan biosythesis

Expression

Median (Tumor)	Median (Normal)	Log2(Fold Change)
52.041	30.500	0.752

Localization for Gene

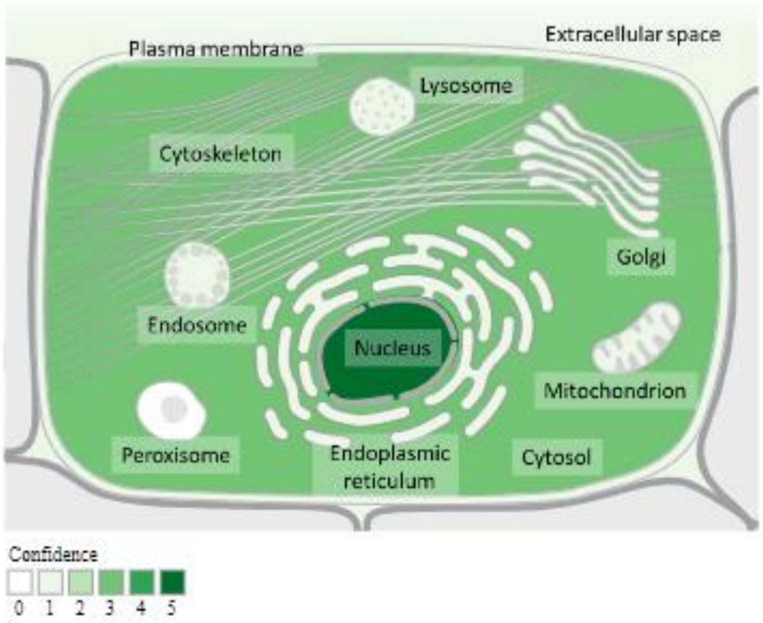

Compartment	Confidence
nucleus	5
cytosol	3
golgi apparatus	1
lysosome	1
endosome	1
endoplasmic reticulum	1
mitochondrion	1
cytoskeleton	1
extracellular	1
plasma membrane	1

Expression in different tissues

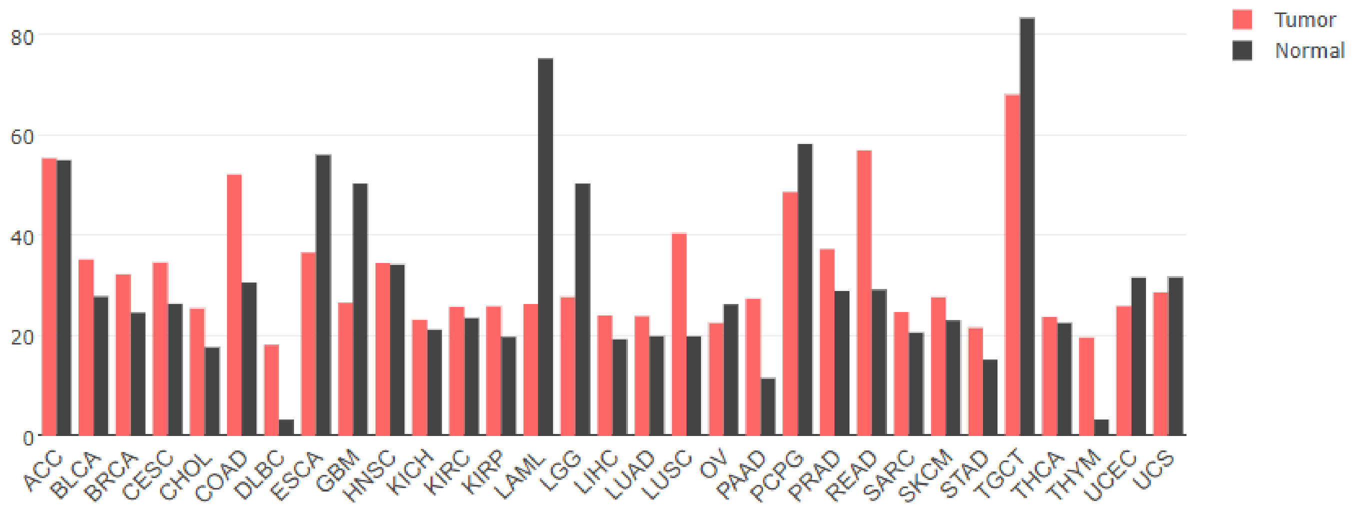

Expression in COAD	Overall Survival

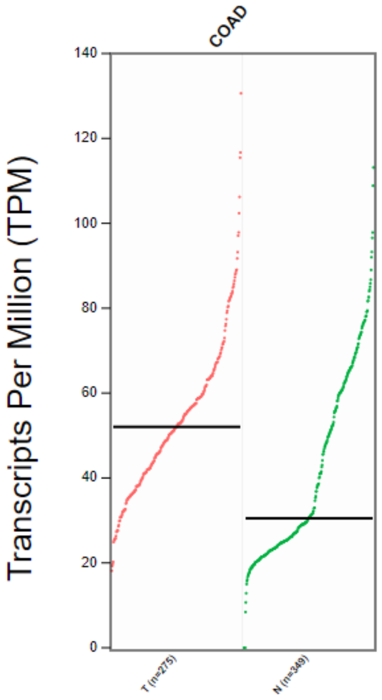

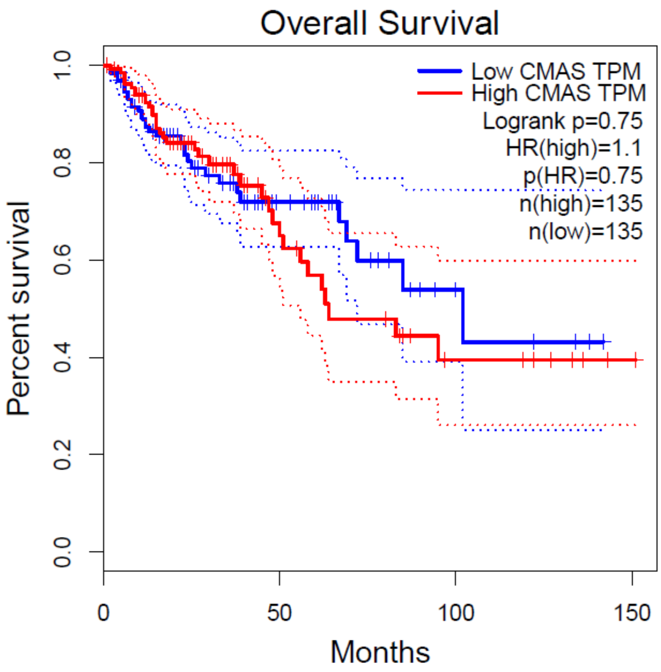

Info	
**NCBI gene ID**	5476
**Official symbol**	CTSA
**Uniprot protein names**	Lysosomal protective protein
**Genecards link**	https://www.genecards.org/cgi-bin/carddisp.pl?gene=CTSA
**Gepia Link**	http://gepia.cancer-pku.cn/detail.php?gene=CTSA
**Proteinatlas Link**	https://www.proteinatlas.org/ENSG00000064601-CTSA/pathology

Drugs from DrugBank

**Name**	**DrugBank ID**	**Type**	**Mechanism**	**Role**
Sofosbuvir	DB08934	approved	substrate	
Tenofovir alafenamide	DB09299	approved	substrate	
Remdesivir	DB14761	approved, investigational	substrate	

Drugs from Genecards

**Name**	**Group**	**Role**	**Mechanism**
Deoxycholic acid	Pharma	Target	Bile Acid;proinflammatory agent
Remdesivir	Pharma	substrate, Enzyme	Antiviral nucleoside analogue
Sofosbuvir	Pharma	substrate, Enzyme	Antiviral agents for chronic HCV infection
Tenofovir alafenamide	Pharma	substrate, Enzyme	HIV reverse transcriptase inhibitor
Boceprevir	Pharma	Inhibition, Inhibitor	HCV protease inhibitor,potent and selective
Telaprevir	Pharma	Inhibition, Inhibitor	HCV NS3-4A protease inhibitor
Odanacatib	Pharma		Cathepsin K,potent and selective, Small Molecule
DiscoveryProbe™ Protease Inhibitor Library	Pharma		A unique collection of 825 protease inhibitors for high throughput screening (HTS) and high content screening (HCS).
E-64-c	Pharma		Inhibitor of cysteine proteinases
Protease Inhibitor Cocktail (100X in DMSO, EDTA plus)	Pharma		Inhibit proteolytic degradation during cell lysis and protein extraction
Protease Inhibitor Cocktail (EDTA-Free, 100X in DMSO)	Pharma		Equivalent to Sigma P8215, for use with fungal and yeast extracts to increase protein stability.
Protease Inhibitor Cocktail (EDTA-Free, 200X in DMSO)	Pharma		Equivalent to Sigma P1860, for use in tissue culture media to prevent proteolytic degradation of secreted proteins.

Protease Inhibitor Cocktail(EDTA-Free,100X in DMSO)	Pharma		Equivalent to Sigma P8340, for use with cell and tissue extracts to increase protein stability
Calpain Inhibitor II, ALLM	Pharma		Calpain inhibitor, Cathepsin inhibitor
E 64d	Pharma		Cysteine protease inhibitor, Cathepsin inhibitor; interferes with autolysosomal digestion
L 006235	Pharma		Cathepsin K inhibitor, Potent cathepsin K inhibitor
SID 26681509	Pharma	Target	Human cathepsin L inhibitor,potent and reversible, Cathepsin L inhibitor

Pathways
Ciliary landscape

Expression		
Median (Tumor)	Median (Normal)	Log2(Fold Change)
97.288	31.899	1.579

Localization for Gene

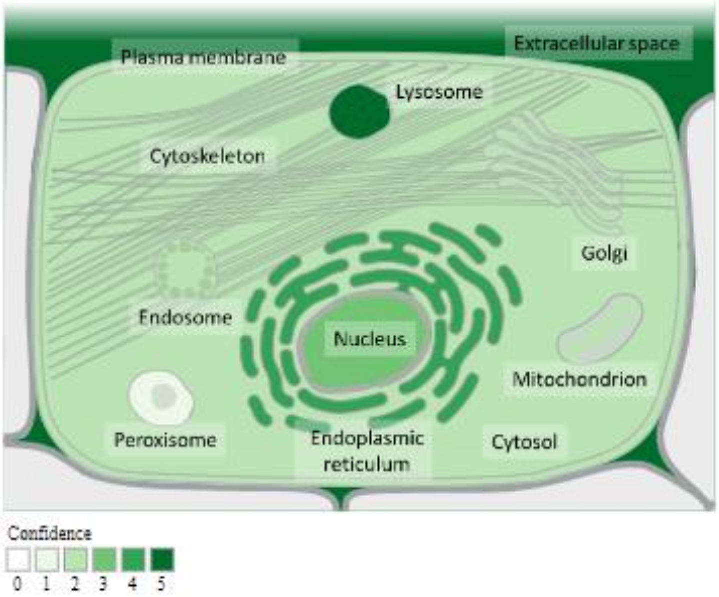

Compartment	Confidence
lysosome	5
extracellular	5
endoplasmic reticulum	4
nucleus	3
golgi apparatus	2
cytosol	2
endosome	2
mitochondrion	2
cytoskeleton	2
plasma membrane	2
peroxisome	1

Expression in different tissues

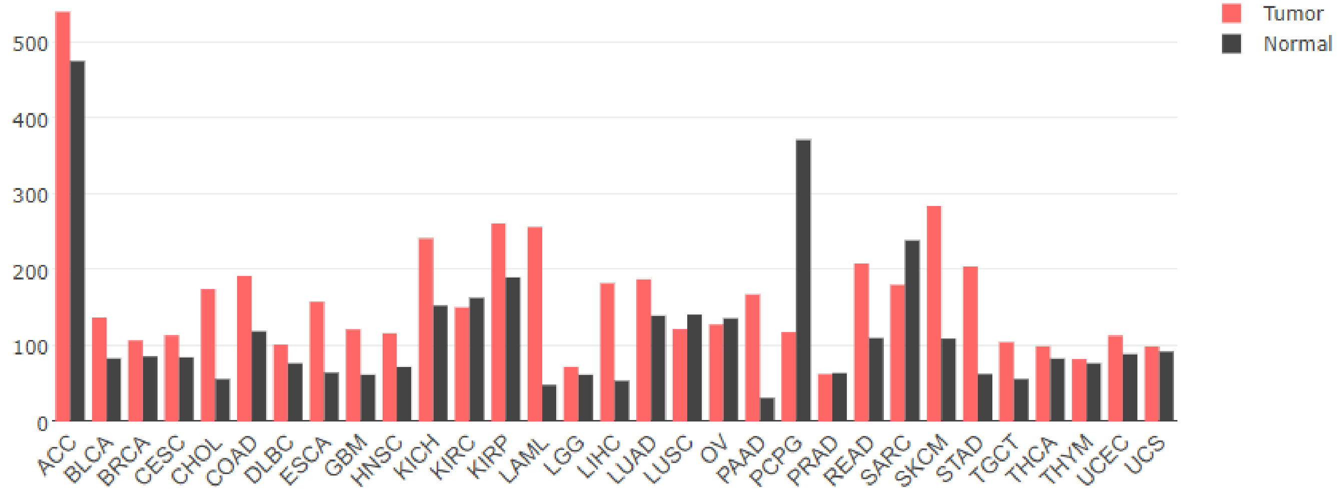

Expression in COAD	Overall Survival
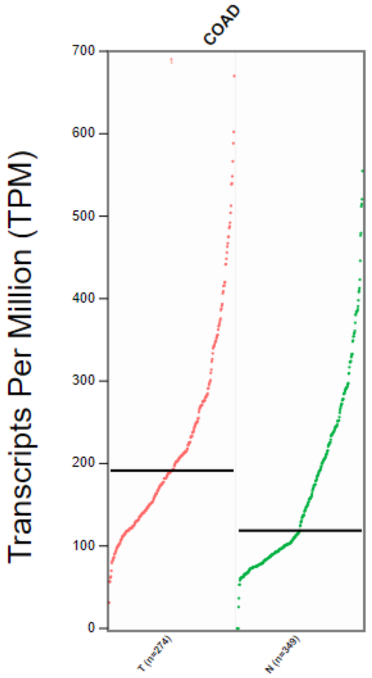	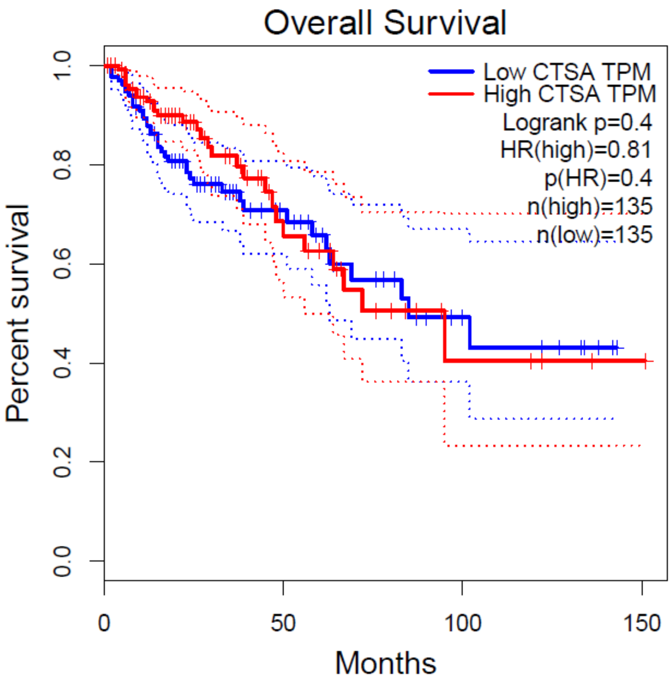

Info	
**NCBI gene ID**	2720
**Official symbol**	GLB1
**Uniprot protein names**	Beta-galactosidase
**Genecards link**	https://www.genecards.org/cgi-bin/carddisp.pl?gene=GLB1
**Gepia Link**	http://gepia.cancer-pku.cn/detail.php?gene=GLB1
**Proteinatlas Link**	https://www.proteinatlas.org/ENSG00000170266-GLB1/pathology

Drugs from DrugBank				
**Name**	**DrugBank ID**	**Type**	**Mechanism**	**Role**
Isopropyl beta-D-thiogalactopyranoside	DB01862		unknown	
D-Galctopyranosyl-1-On	DB01885		unknown	
1-O-[O-Nitrophenyl]-Beta-D-Galactopyranose	DB01920		unknown	
2-deoxy-2-fluoro-β-D-galactose	DB02228		unknown	
(5R,6S,7S,8S)-5-hydroxymethyl-6,7,8-trihydroxy-tetrazolo[1,5-A]piperidine	DB02294		unknown	
4-nitrophenyl-beta-D-galactoside	DB02632		unknown	
Allolactose	DB04116		unknown	
2-Fluoro-2-Deoxy-Beta-D-Galactopyranosyl-Beta-D-Glucopyranose	DB04155		unknown	
2-Deoxy-alpha-D-galactopyranose	DB04382		unknown	
Lactose	DB04465		unknown	
S,S-(2-Hydroxyethyl)Thiocysteine	DB04530		unknown	
Tyrothricin	DB13503		inhibitor	Very weak inhibition.

Drugs from Genecards			
**Name**	**Group**	**Role**	**Mechanism**
Lactose	Pharma	Target	
Beta-D-Glucose	Pharma		Simple sugar (monosaccharide)
Glycerin	Pharma		
Phosphoric acid	Pharma		
Water	Pharma		
Ribonic acid gamma lactone	Pharma		An inhibitor of E. coli β-galactosidase
Lactose, anhydrous			

Pathways
Ciliary landscape
Degradation pathway of sphingolipids, including diseases
Ectoderm differentiation
Glycosaminoglycan degradation
Sphingolipid metabolism in senescence

Expression		
Median (Tumor)	Median (Normal)	Log2(Fold Change)
55.799	21.769	1.319

Localization for Gene

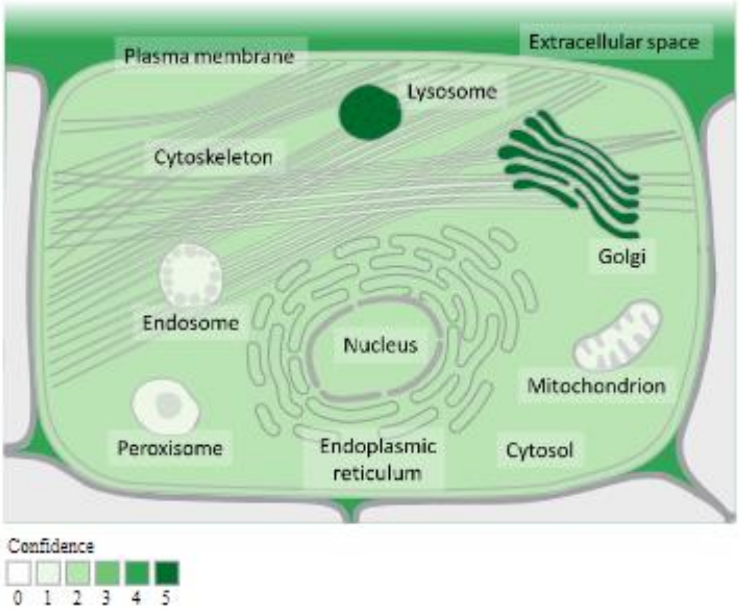

Compartment	Confidence
golgi apparatus	5
lysosome	5
extracellular	4
cytosol	2
endoplasmic reticulum	2
nucleus	2
plasma membrane	2
endosome	1
peroxisome	1
mitochondrion	1
cytoskeleton	1

Expression in different tissues

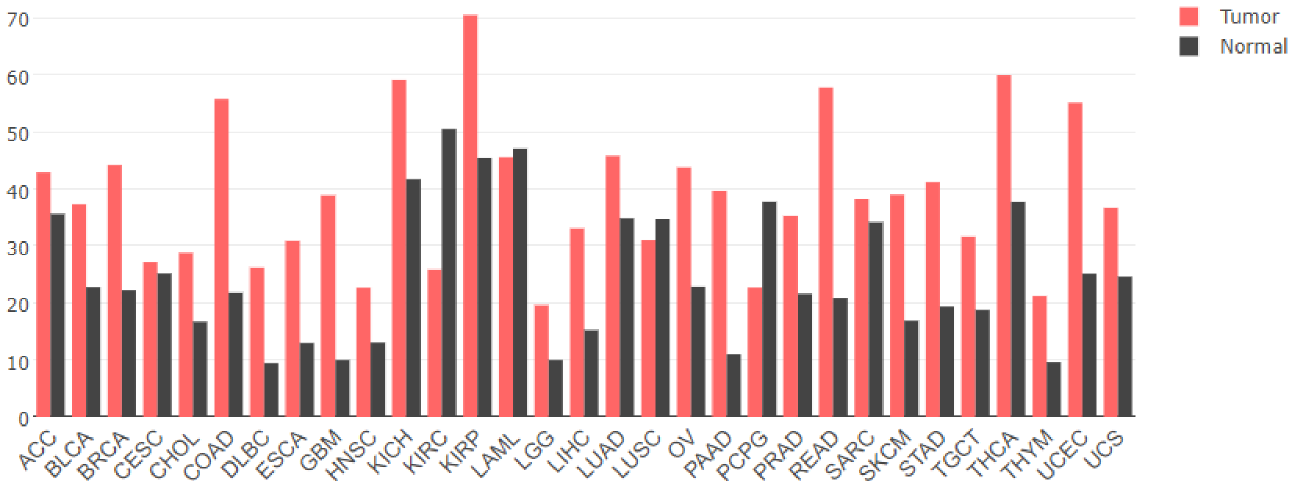

Expression in COAD	Overall Survival

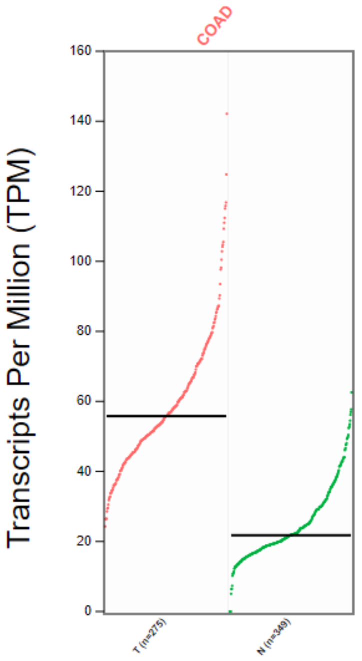

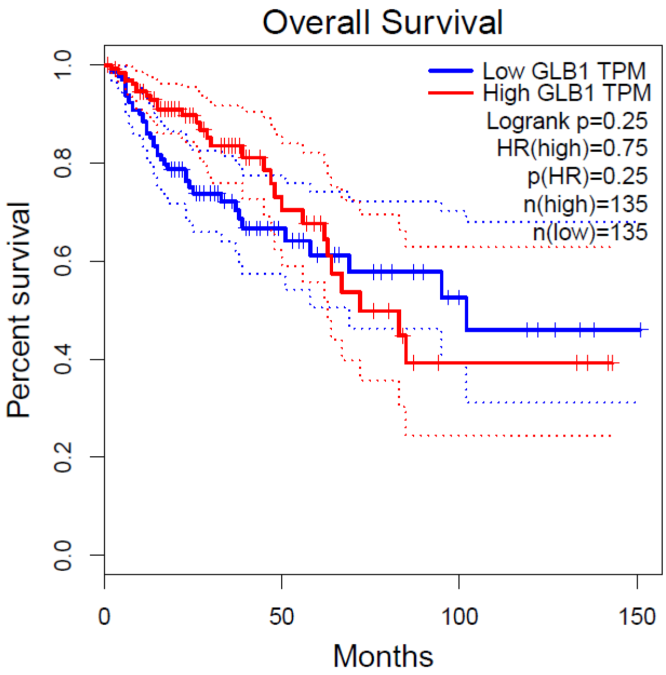

Info	
**NCBI gene ID**	2588
**Official symbol**	GALNS
**Uniprot protein names**	N-acetylgalactosamine-6-sulfatase
**Genecards link**	https://www.genecards.org/cgi-bin/carddisp.pl?gene=GALNS
**Gepia Link**	http://gepia.cancer-pku.cn/detail.php?gene=GALNS
**Proteinatlas Link**	https://www.proteinatlas.org/ENSG00000141012-GALNS/pathology

Drugs from DrugBank				
**Name**	**DrugBank ID**	**Type**	**Mechanism**	**Role**
Chondroitin sulfate	DB09301		approved, investigational, nutraceutical	substrate

Drugs from Genecards			
**Name**	**Group**	**Role**	**Mechanism**
Chondroitin sulfate	Nutra	substrate, Enzyme	
Elosulfase alfa	Pharma		
Morphine	Pharma		Small Molecule, Analgesics, Opioid
Water	Pharma		
Sulfuric acid	Pharma		
Phenyl hydrogen sulfate	Pharma		Belongs to the class of organic compounds known as phenylsulfates
Anesthetics	Pharma		
Pharmaceutical Solutions	Pharma		
D-+-Galactosamine	Pharma		
N-acetyl D-galactosamine	Pharma		An endogenous metabolite
Calcium	Nutra	substrate, Enzyme	

Pathways
Glycosaminoglycan degradation

Expression		
Median (Tumor)	Median (Normal)	Log2(Fold Change)
?	?	?

Localization for Gene

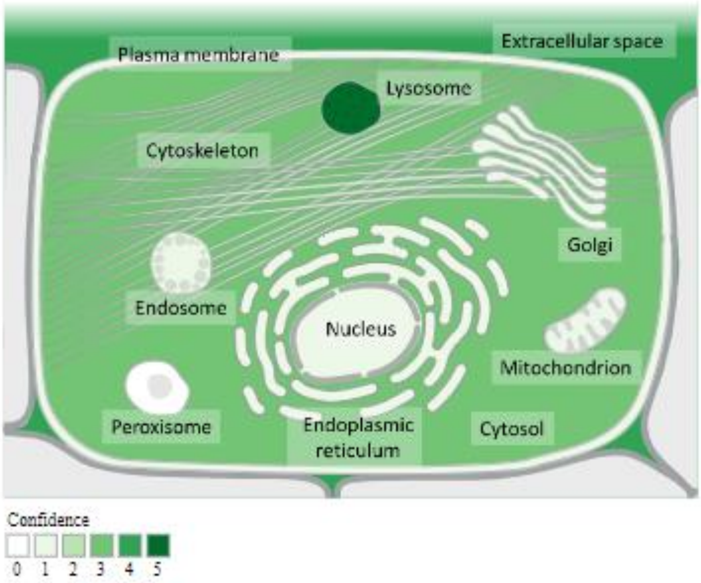

Compartment	Confidence
lysosome	5
extracellular	4
cytosol	3
golgi apparatus	1
endosome	1
endoplasmic reticulum	1
nucleus	1
mitochondrion	1
cytoskeleton	1
plasma membrane	1
peroxisome	0

Expression in different tissues

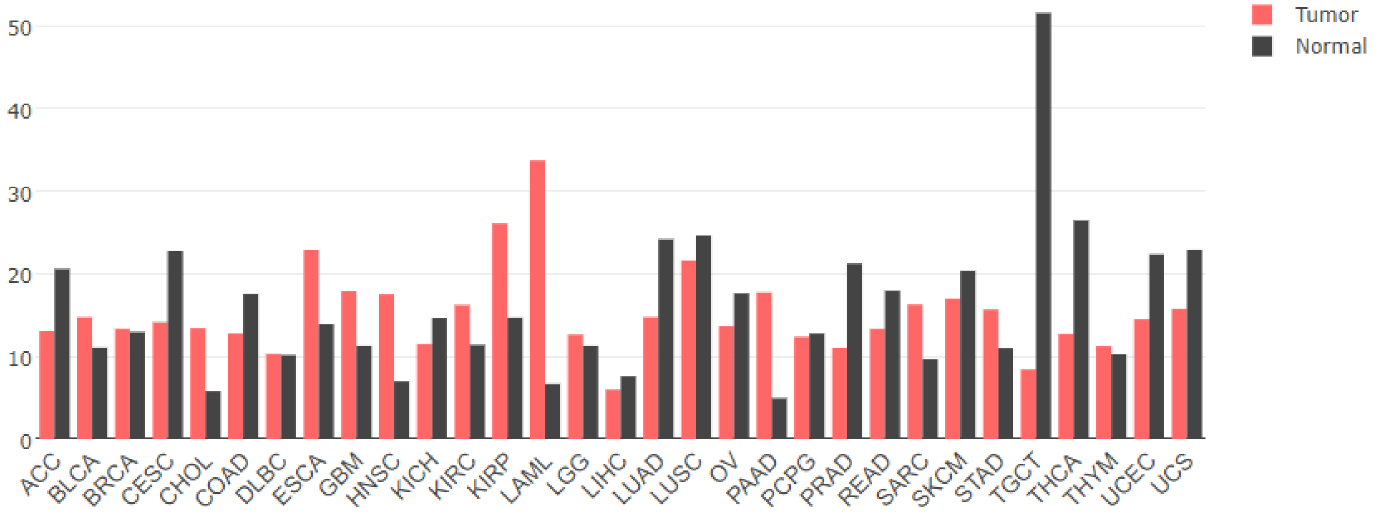

Expression in COAD	Overall Survival

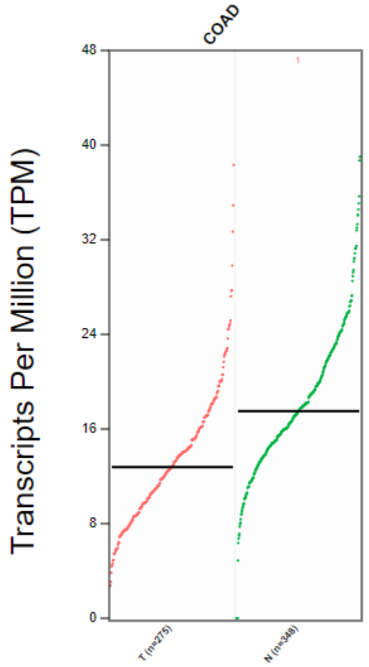

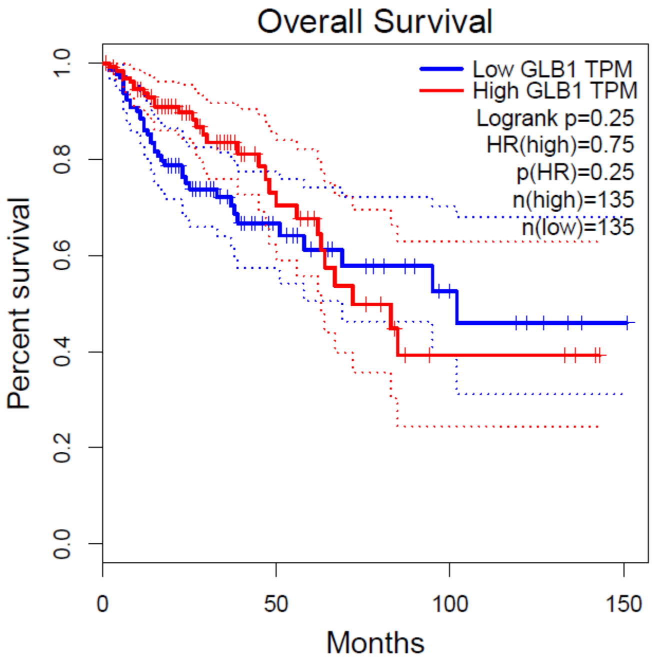

Info	
**NCBI gene ID**	4758
**Official symbol**	NEU1
**Uniprot protein names**	Sialidase-1
**Genecards link**	https://www.genecards.org/cgi-bin/carddisp.pl?gene=NEU1
**Gepia Link**	http://gepia.cancer-pku.cn/detail.php?gene=NEU1
**Proteinatlas Link**	https://www.proteinatlas.org/ENSG00000204386-NEU1/pathology

Drugs from DrugBank				
**Name**	**DrugBank ID**	**Type**	**Mechanism**	**Role**
Oseltamivir	DB00198		approved	inhibitor
Acetylsalicylic acid	DB00945		approved, vet_approved	inhibitor
Celecoxib	DB00482		approved, investigational	inhibitor

Drugs from Genecards			
**Name**	**Group**	**Role**	**Mechanism**
Oseltamivir	Pharma	inhibitor, Target	Potent and selective inhibitor of the neuraminidase, inhibitor of influenza neuraminidase
Peramivir	Pharma		Aeuraminidase inhibitor;antiviral drug
Zanamivir	Pharma		Influenza A/B virus neuraminidases inhibitor
Acetylsalicylic acid	Pharma	inhibitor, Target	Cyclooxygenase (COX) inhibitor, Anticoagulants, Anti-Inflammatory Agents, Non-Steroidal
Celecoxib	Pharma	inhibitor, Target	Selective cyclooxygenase-2 (COX-2) inhibitor, Small Molecule
Water	Pharma		
4-acetamido-	Pharma	Target	
Aceneuramic acid	Pharma		Human Endogenous Metabolite
Arbidol HCl	Pharma		Antiviral chemical agent
Dryocrassin ABBA	Pharma		
Filixic acid ABA	Pharma		
Peramivir Trihydrate	Pharma		potent, specific influenza viral neuraminidase inhibitor
X-NeuNAc	Pharma		Subtrate for chromogeneic assay of neuraminidase activity
Name	Group	Role	Mechanism of Action
Acetylcholine	Pharma		
Acyclovir	Pharma		Antiviral agent

Amantadine	Pharma		
Benzoic acid	Pharma		Organic aromatic monocarboxylic acid
Carbamoylcholine	Pharma	inhibitor, Target	Small Molecule, Antiglaucomic Agents

Pathways
Degradation pathway of sphingolipids, including diseases

Expression		
Median (Tumor)	Median (Normal)	Log2(Fold Change)
?	?	?

Localization for Gene

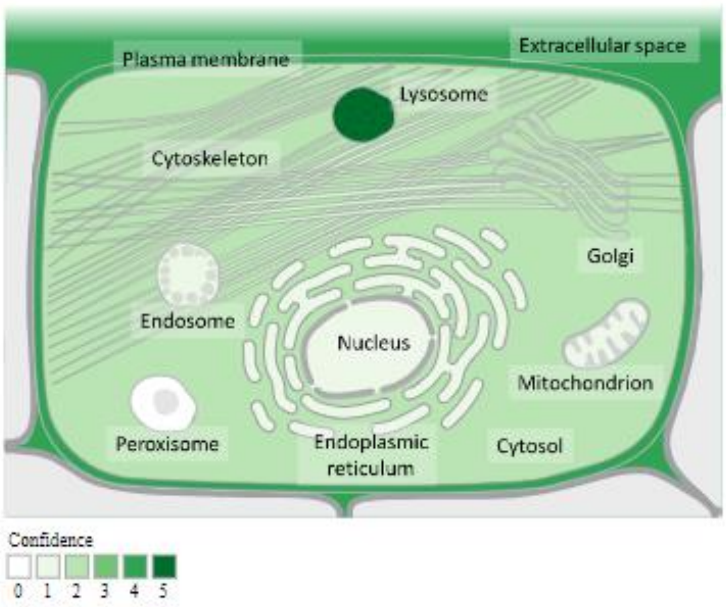

Compartment	Confidence
lysosome	5
extracellular	4
plasma membrane	4
golgi apparatus	2
cytosol	2
endosome	1
endoplasmic reticulum	1
nucleus	1
mitochondrion	1
cytoskeleton	1

Expression in different tissues

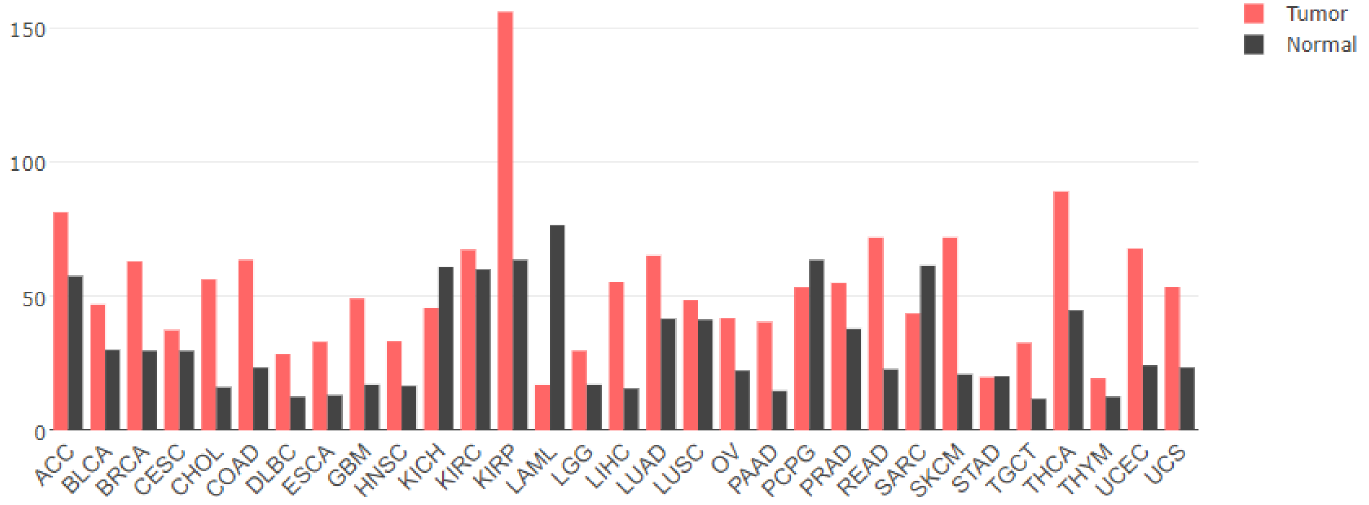

Expression in COAD	Overall Survival

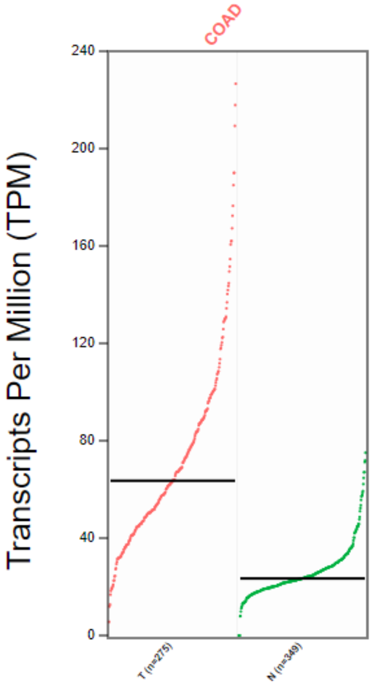

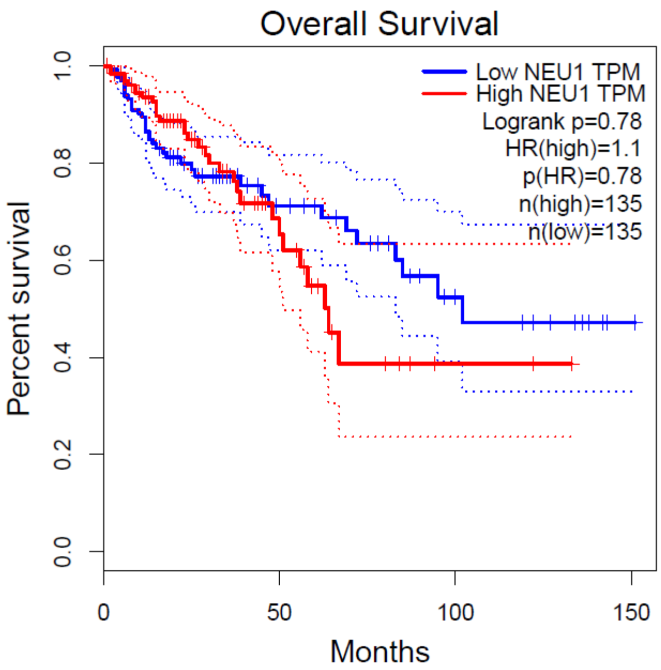

Info	
**NCBI gene ID**	9583
**Official symbol**	ENTPD4
**Uniprot protein names**	Ectonucleoside triphosphate diphosphohydrolase 4
**Genecards link**	https://www.genecards.org/cgi-bin/carddisp.pl?gene=NEU1
**Gepia Link**	http://gepia.cancer-pku.cn/detail.php?gene=NEU1
**Proteinatlas Link**	https://www.proteinatlas.org/ENSG00000204386-NEU1/pathology

Drugs from DrugBank				
**Name**	**DrugBank ID**	**Type**	**Mechanism**	**Role**

Drugs from Genecards			
**Name**	**Group**	**Role**	**Mechanism**
Magnesium	Pharma		
Phosphoric acid	Pharma		
Water	Pharma		
Cytidine-5'-Diphosphate	Pharma		
Guanosine-5'-Diphosphate	Pharma		
Guanosine-5'-Monophosphate	Pharma		
Guanosine-5'-Triphosphate	Pharma		
Inosinic Acid	Pharma		
Thymidine-5'-Diphosphate	Pharma		
Uridine monophosphate	Pharma		
Uridine-5'-Diphosphate	Pharma		
Adenosine disphosphate	Pharma		central component of energy storage, metabolism, and signal transduction in vivo;agonist of purinergic receptors
Calcium	Nutra		

Pathways from Reactome
Metabolism
Metabolism of nucleotides
Nucleotide catabolism
Phosphate bond hydrolysis by NTPDase proteins

Expression		
Median (Tumor)	Median (Normal)	Log2(Fold Change)
12.140	20.100	-0.683

Localization for Gene

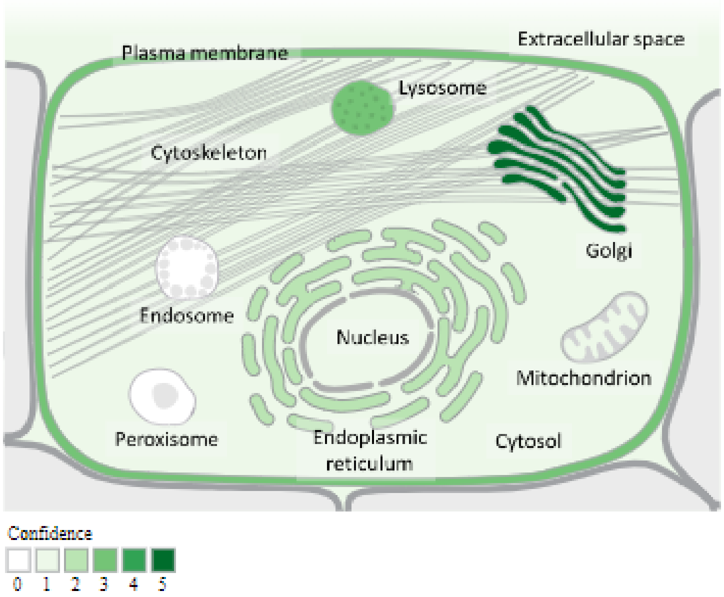

Compartment	Confidence
golgi apparatus	5
lysosome	3
plasma membrane	3
endoplasmic reticulum	2
cytosol	1
nucleus	1
mitochondrion	1
cytoskeleton	1
extracellular	1

Expression in different tissues

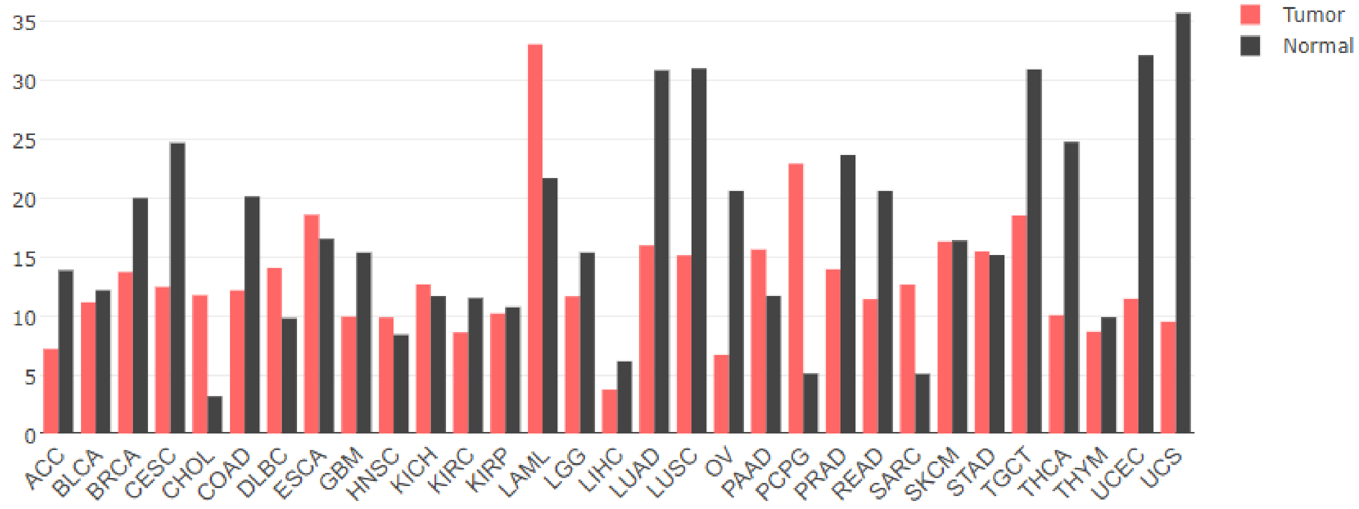

Expression in COAD	Overall Survival

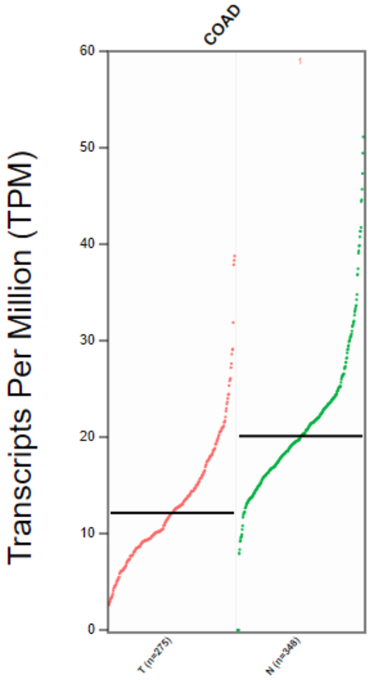

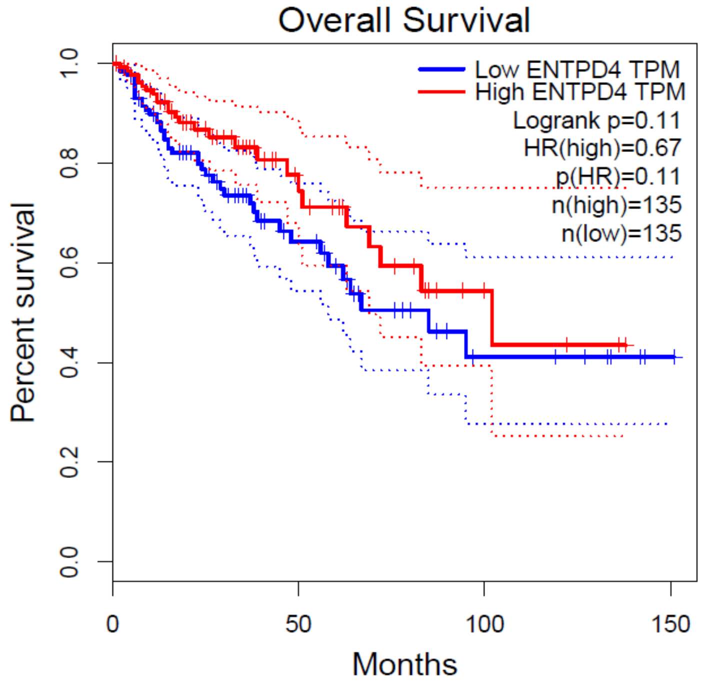

Info	
**NCBI gene ID**	7355
**Official symbol**	SLC35A2
**Uniprot protein names**	UDP-galactose translocator
**Genecards link**	https://www.genecards.org/cgi-bin/carddisp.pl?gene=SLC35A2
**Gepia Link**	http://gepia.cancer-pku.cn/detail.php?gene=SLC35A2
**Proteinatlas Link**	https://www.proteinatlas.org/ENSG00000102100-SLC35A2/pathology

Drugs from DrugBank				
**Name**	**DrugBank ID**	**Type**	**Mechanism**	**Role**

Drugs from Genecards			
**Name**	**Group**	**Role**	**Mechanism**

Pathways from Reactome
Defective SLC35A2 causes congenital disorder of glycosylation 2M (CDG2M)
Disease
Disorders of transmembrane transporters
SLC transporter disorders
SLC-mediated transmembrane transport
Transport of nucleotide sugars
Transport of small molecules
Transport of vitamins, nucleosides, and related molecules

Expression		
Median (Tumor)	Median (Normal)	Log2(Fold Change)
36.339	12.45	1.473

Localization for Gene

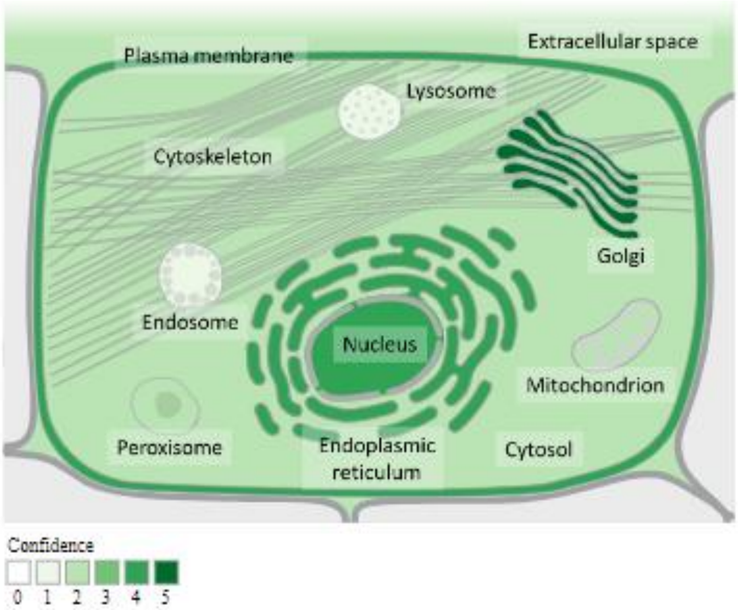

Compartment	Confidence
golgi apparatus	5
endoplasmic reticulum	4
nucleus	4
plasma membrane	4
cytosol	2
peroxisome	2
mitochondrion	2
cytoskeleton	2
extracellular	2
lysosome	1
endosome	1

Expression in different tissues

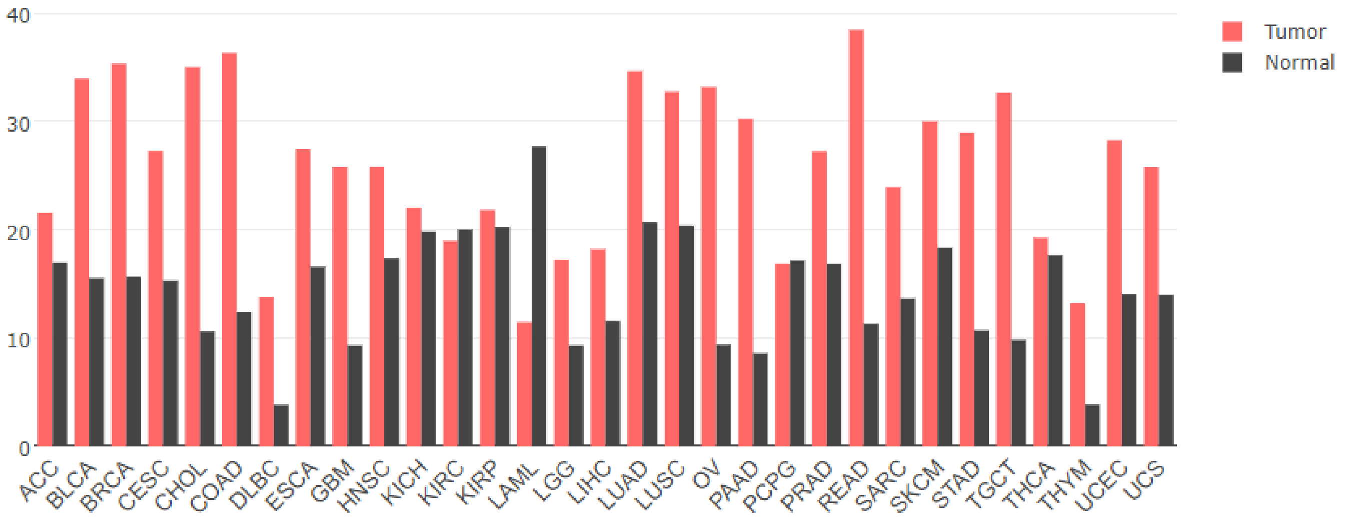

Expression in COAD	Overall Survival

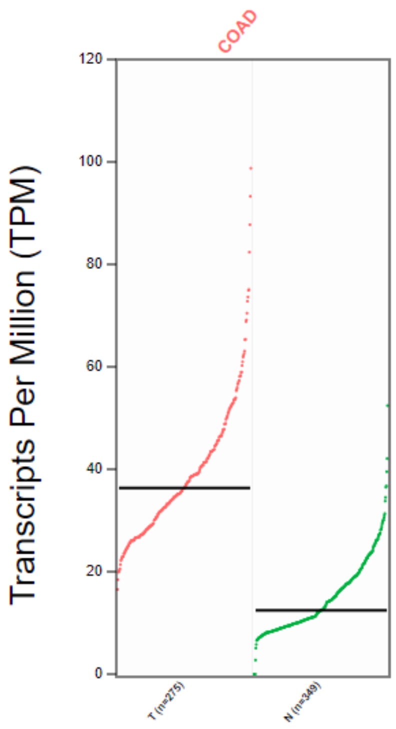

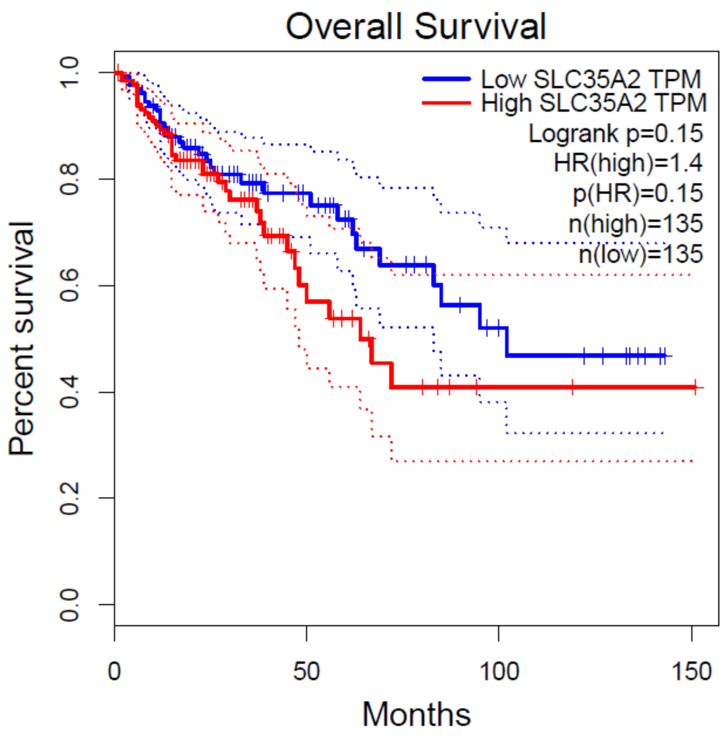

Info	
**NCBI gene ID**	11046
**Official symbol**	SLC35D2
**Uniprot protein names**	UDP-N-acetylglucosamine/UDP-glucose/GDP-mannose transporter
**Genecards link**	https://www.genecards.org/cgi-bin/carddisp.pl?gene=SLC35D2
**Gepia Link**	http://gepia.cancer-pku.cn/detail.php?gene=SLC35D2
**Proteinatlas Link**	https://www.proteinatlas.org/ENSG00000130958-SLC35D2/pathology

Drugs from DrugBank				
**Name**	**DrugBank ID**	**Type**	**Mechanism**	**Role**

Drugs from Genecards			
**Name**	**Group**	**Role**	**Mechanism**

Pathways from Reactome
Glycosaminoglycan metabolism
Heparan sulfate/heparin (HS-GAG) metabolism
HS-GAG biosynthesis
Keratan sulfate biosynthesis
Keratan sulfate/keratin metabolism
Metabolism
Metabolism of carbohydrates
SLC-mediated transmembrane transport
Transport of nucleotide sugars
Transport of small molecules
Transport of vitamins, nucleosides, and related molecules

Expression		
Median (Tumor)	Median (Normal)	Log2(Fold Change)
46.84	25.539	0.85

Localization for Gene

Subcellular locations from COMPARTMENTS

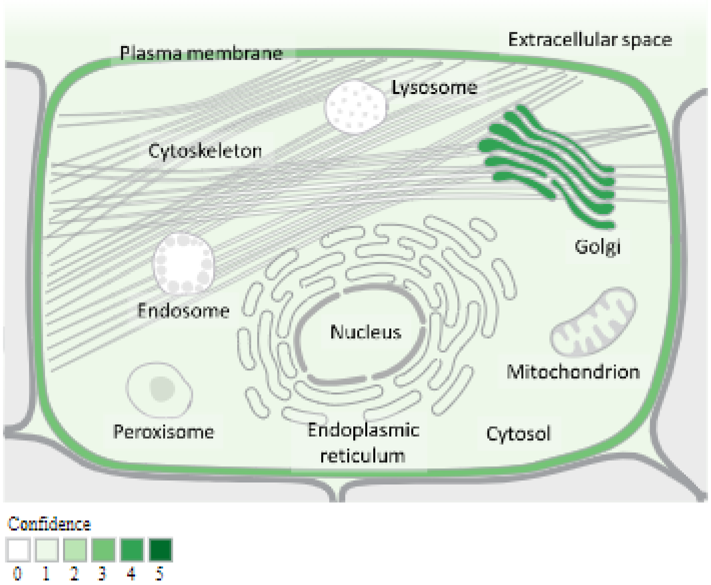

Compartment	Confidence
golgi apparatus	4
plasma membrane	3
cytosol	1
endoplasmic reticulum	1
nucleus	1
peroxisome	1
mitochondrion	1
extracellular	1

Expression in different tissues

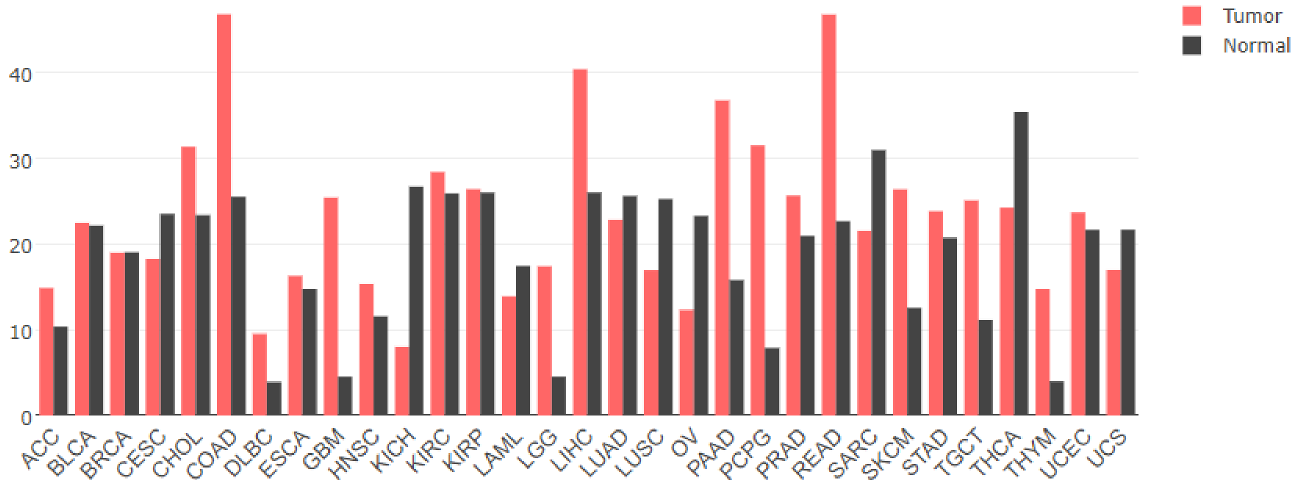

Expression in COAD	Overall Survival

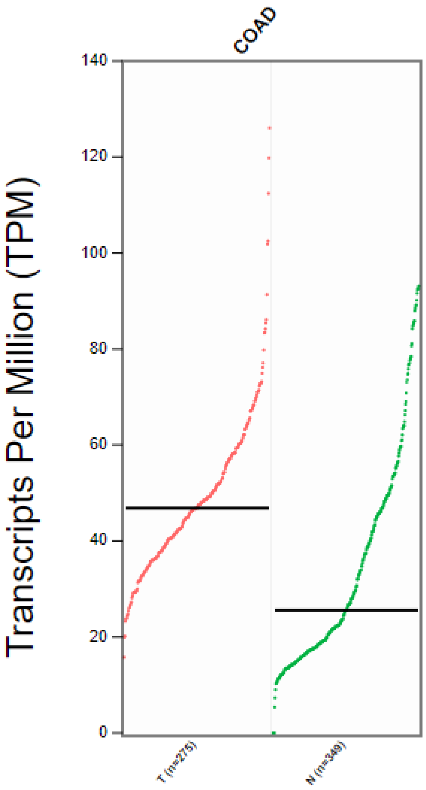

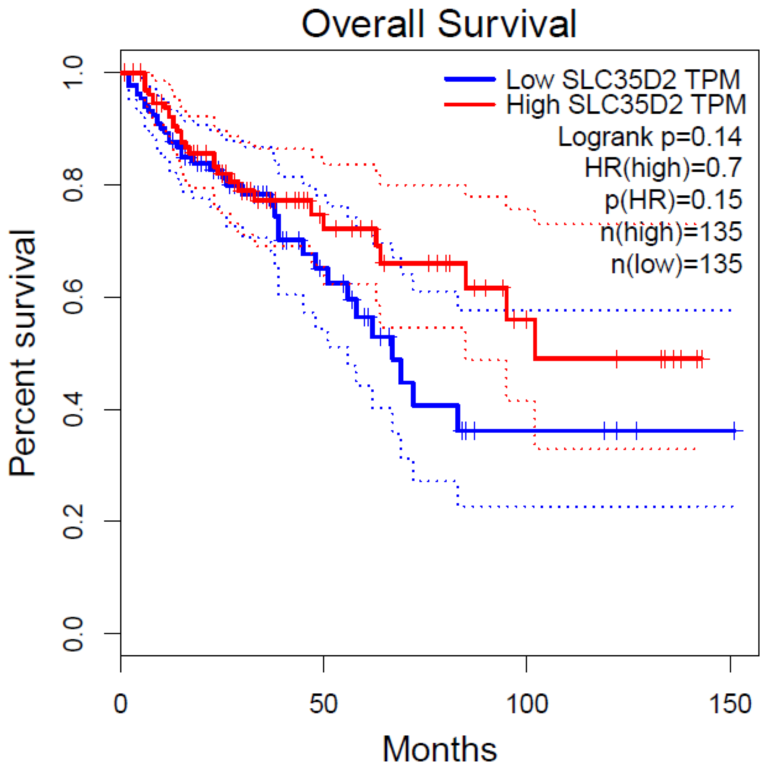

References

1. GeneCards. https://www.genecards.org/.
2. Gepia. http://gepia.cancer-pku.cn/index.html.
3. Proteinatlas. https://www.proteinatlas.org/.
