# Supplementary material for: Higher order synthetic lethals are keys to minimize cancer treatment effects on non-tumor cells
Source: PLoS One. 2026 Apr 15;21(4):e0342199. doi: 10.1371/journal.pone.0342199 (PMC13082654; doi:10.1371/journal.pone.0342199)
Supplement: S3 File — (PDF) [file pone.0342199.s003.pdf]

Supplymentary file for: The interaction graphs for LIHC and KIRC *strictly-selective* targets

Short title: Cancer treatment using higher order synthetic lethals

Mehdi Dehghan Manshadi, Payam Setoodeh, Amin Ramezani, Amin Reza Rajabzadeh, Habil Zare

Correspondence should be addressed to Habil Zare: zare@uthscsa.edu and Payam Setoodeh: payamst@shirazu.ac.ir

General information: The relationship between different genes in obtained solutions for LIHC and KIRC is visualized in S1 Fig and S2 Fig based on their co-presence in each SL set. Single essentials are shown in red, double SLs in purple and connected by wavy lines, triple SLs in green and connected by dashed lines, and quadruple SLs in blue and connected by solid lines

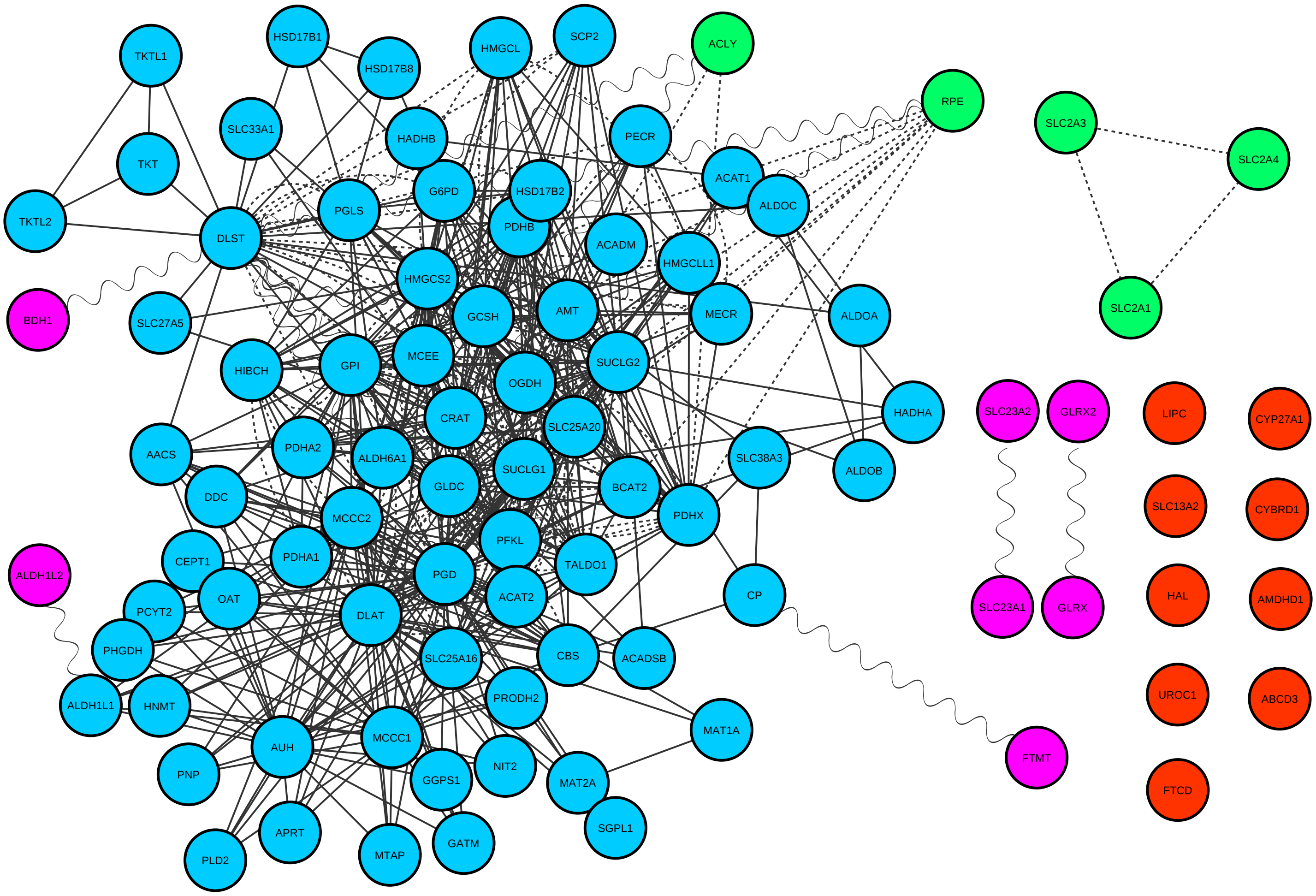

S1 Fig. The relation between different genes of identified strictly-selective solutions for LIHC.

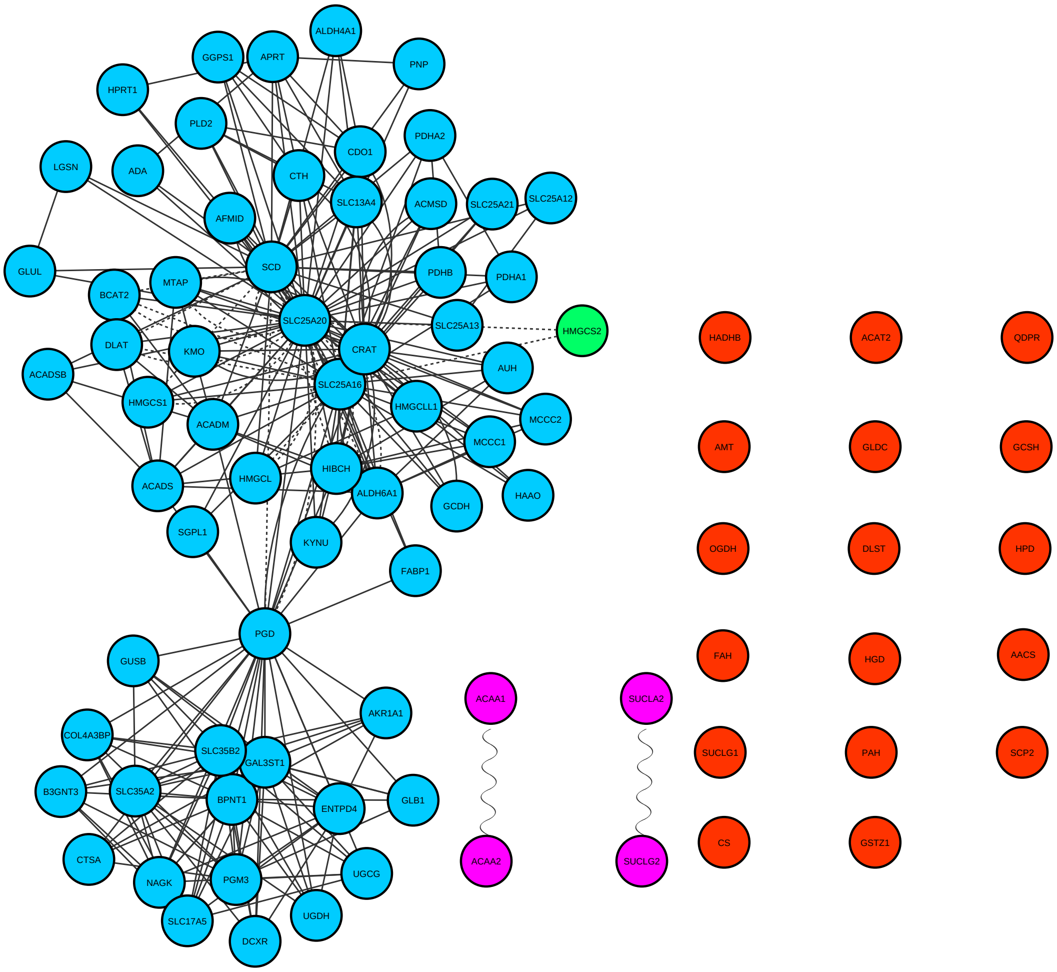

S2 Fig. The relation between different genes of identified strictly-selective solutions for KIRC.
